# AMPK activator-treated human cardiac spheres enhance maturation and enable pathological modeling

**DOI:** 10.1186/s13287-023-03554-7

**Published:** 2023-11-08

**Authors:** Dong Li, Lawrence C. Armand, Fangxu Sun, Hyun Hwang, David Wolfson, Antonio Rampoldi, Rui Liu, Parvin Forghani, Xin Hu, Wen-Mei Yu, Cheng-Kui Qu, Dean P. Jones, Ronghu Wu, Hee Cheol Cho, Joshua T. Maxwell, Chunhui Xu

**Affiliations:** 1 Department of Pediatrics, Emory University School of Medicine and Children’s Healthcare of Atlanta, Atlanta, GA USA; 2 School of Chemistry and Biochemistry, Georgia Institute of Technology, Atlanta, GA 30332 USA; 3 Wallace H. Coulter Department of Biomedical Engineering, Georgia Institute of Technology and Emory University, Atlanta, GA USA; 4 Department of Medicine, Emory University School of Medicine, Atlanta, GA USA

**Keywords:** AMPK, Cardiomyocytes, Maturation, Metabolic regulation, Pathological modeling, Stem cells

## Abstract

**Background:**

Cardiac pathological outcome of metabolic remodeling is difficult to model using cardiomyocytes derived from human-induced pluripotent stem cells (hiPSC-CMs) due to low metabolic maturation.

**Methods:**

hiPSC-CM spheres were treated with AMP-activated protein kinase (AMPK) activators and examined for hiPSC-CM maturation features, molecular changes and the response to pathological stimuli.

**Results:**

Treatment of hiPSC-CMs with AMPK activators increased ATP content, mitochondrial membrane potential and content, mitochondrial DNA, mitochondrial function and fatty acid uptake, indicating increased metabolic maturation. Conversely, the knockdown of AMPK inhibited mitochondrial maturation of hiPSC-CMs. In addition, AMPK activator-treated hiPSC-CMs had improved structural development and functional features—including enhanced Ca^2+^ transient kinetics and increased contraction. Transcriptomic, proteomic and metabolomic profiling identified differential levels of expression of genes, proteins and metabolites associated with a molecular signature of mature cardiomyocytes in AMPK activator-treated hiPSC-CMs. In response to pathological stimuli, AMPK activator-treated hiPSC-CMs had increased glycolysis, and other pathological outcomes compared to untreated cells.

**Conclusion:**

AMPK activator-treated cardiac spheres could serve as a valuable model to gain novel insights into cardiac diseases.

**Supplementary Information:**

The online version contains supplementary material available at 10.1186/s13287-023-03554-7.

## Background

Heart disease is the number one cause of death and has become a significant issue to public health. Due to differences in physiology among species, animal models have limitations in modeling human pathological conditions—thus encouraging the exploration of more human relevant resources. Following the breakthrough in the development of human-induced pluripotent stem cells (hiPSCs), hiPSC-derived cardiomyocytes (hiPSC-CMs) have emerged as an unprecedented opportunity to overcome the limitations of current models in modeling human diseases and drug discovery. However, compared with primary human cardiomyocytes, hiPSC-CMs resemble fetal cardiomyocytes more closely in structure and function, imposing limitations in the use of hiPSC-CMs [1, 2]. Therefore, further enhancement of hiPSC-CMs maturity in a scalable manner has become paramount to improve applications of these cells. We and others have demonstrated that combining HIF-1α inhibition with molecules that target key pathways involved in energy metabolism could significantly promote hiPSC-CMs to be both structurally and functionally more mature [3, 4]. These exciting findings proved the feasibility of improving hiPSC-CM maturity through metabolic regulation of certain signaling pathways.

Metabolic remodeling on increasing fatty acid oxidation (FAO) for energy production is a hallmark of cardiomyocyte maturation during heart development [1, 2]. More than half of the cellular ATP utilized in a healthy mature heart is produced from FAO [5, 6]. On the other hand, under pathological conditions, metabolic switch from FAO to glycolysis in the heart is a key pathological outcome [7]. Modeling this pathological outcome associated with a metabolic switch to glycolysis has been challenging in hiPSC-CMs since these immature cells mostly rely on glycolysis for energy production and have low levels of FAO.

We hypothesized that activation of adenosine 5′-monophosphate (AMP)-activated protein kinase (AMPK) could potentially be a key regulator in the transition of hiPSC-CMs from immature phenotype to a more mature stage and that such maturation treatment would facilitate the use of hiPSC-CMs in pathological modeling. AMPK is a serine/threonine heterotrimeric kinase that plays an important role in the regulation of glucose, lipid and protein metabolism in the heart [8]. In cardiomyocytes, AMPK functions as a key metabolic sensor of intracellular energy and plays a direct role in controlling a wide variety of cellular processes that can influence cardiomyocyte function and maturation. AMPK expression and enzymatic activity are upregulated during the development of newborn heart, leading to decreased glycolysis and increased FAO [9]. In hiPSC-CMs, activated AMPK caused by PRAG2 cardiomyopathy mutations results in increased mitochondrial biogenesis and cardiac forces [10]. However, further study is needed to examine whether pharmacological agent-induced AMPK activation can induce hiPSC-CM maturation, and whether AMPK-modulated hiPSC-CMs are suitable for pathological modeling.

In this study, we developed an efficient strategy to screen for optimal AMPK activators and their doses for promoting metabolic maturation of hiPSC-CMs. We then further evaluated the effect of AMPK activators on the metabolic, structural and functional maturation of 3D hiPSC-CMs. The results demonstrated that AMPK activation significantly improved mitochondrial function, FAO, structural development, calcium handling, electrophysiological properties and contractility index. Transcriptomic, proteomic and metabolomic profiling of AMPK activator-treated hiPSC-CMs revealed that these cells had a distinct expression profile with a subset of genes, proteins and metabolites that resemble the molecular signature of mature cardiomyocytes. Furthermore, we discovered that AMPK activator-treated hiPSC-CMs responded to pathological stimuli, which was not achievable in untreated immature hiPSC-CMs, highlighting the benefit of AMPK-modulated maturation.

## Methods

Additional file 1: Methods contain culture of human-induced pluripotent stem cell lines (IMR90 and SCVI-273), cardiac differentiation, formation of cardiac spheres, AMPK activators dose screening, immunocytochemical analysis, high-content imaging analysis by ArrayScan, quantification of mitochondrial DNA content, qRT-PCR analysis, flow cytometry, detection of ATP content, mitochondrial membrane potential measurement, measurement of sarcomere length, cell area, cell perimeter and length/width ratio, fatty acid uptake assay, glucose uptake assay, calcium imaging, measurement of action potential using FluoVolt, contractility analysis, RNA-sequencing (RNA-seq) analysis, proteomics analysis, high-resolution metabolomics, cell viability assay, Nile red staining and detection of cell apoptosis.

### Treatments of cardiac spheres with AMPK activators

Cardiac spheres were generated on differentiation day 5 and maintained in RPMI + B27 medium containing insulin until day 14. On day 14, the medium was replaced with the maturation medium supplemented with following (1) vehicle (DMSO) control; (2) EX229 at 10 µM (E10); (3) EX229 at 50 µM (E50); (4) A-769662 at 100 µM (A100) and (5) A-769662 at 200 µM (A200). Cardiac spheres were treated with AMPK activators for 7 days or 14 days prior to be harvested for outcome analyses.

### Seahorse XF24 metabolic flux analysis

Mitochondrial function was analyzed using the XF Cell Mito Stress Kit (Agilent Technologies, Santa Clara, CA). Following treatment with AMPK activators or DMSO, cardiac spheres were dissociated into single cells using 0.25% trypsin/EDTA and plated onto a Seahorse XF-24 cell culture plate (Agilent Technologies, Santa Clara, CA) coated with Matrigel (1:50) at a density of 1.2 × 10^5^ cells/well with 10-µM Rock inhibitor Y-276322. After 24 h, the maturation medium was refreshed, and cells were maintained in the maturation medium for 1–2 additional days. One hour before the assay, the cells were washed with base medium (XF DMEM medium + 5-mM glucose + 2-mM L-glutamine + 0.5-mM sodium pyruvate) and incubated in 525-µL base medium/well at 37 °C in a non-CO_2_ incubator. Oligomycin (2 µM), carbonyl cyanide p-(trifluoromethoxy) phenylhydrazone (FCCP, 1 µM) and rotenone (0.5 µM) + antimycin A (0.5 µM) were diluted in base medium and sequentially injected into each well during the measurements of oxygen consumption (OCR). The results were normalized to 1x10^4^ cells.

FAO and glycolysis were determined using a Seahorse XF 24 analyzer (Agilent Technologies, Santa Clara, CA). Etomoxir (ETO, Sigma-Aldrich, St. Louis, MO), a specific carnitine palmitoyltransferase 1 (CPT1) inhibitor and 2-deoxyglucose (2-DG, Agilent Technologies, Santa Clara, CA), a glucose analog that inhibits glycolysis, were diluted in base medium to gain the working concentration of 100 µM for ETO and 50 mM for 2-DG and sequentially injected into each well during the measurements. The levels of FAO were then calculated based on the difference in the OCR before and after the addition of ETO. The levels of glycolysis were calculated based on the difference in the extracellular acidification rate (ECAR) before and after the addition of 2-DG. The results were normalized to 1x10^5^ cells. Measurement of metabolic fluxes has accuracies comparable to those obtained with the radiometric measurement of FAO [11].

Glycolytic function in the cells after pathological modeling was analyzed using the XF Glycolysis Stress Test Kit (Agilent Technologies, Santa Clara, CA). One hour before the assay, the cells were washed with base medium (XF DMEM medium + 2-mM L-glutamine) and incubated in 525-µL base medium/well at 37 °C in a non-CO_2_ incubator. Glucose (10 mM), oligomycin (1 µM) and 2-DG (50 mM) diluted in base medium were sequentially injected into each well during the measurements. The results were normalized to 1x10^4^ cells.

### AMPK inhibition using Compound C

On differentiation day 14, cardiac spheres were treated with AMPK activators with or without 5-µM Compound C (AMPK inhibitor; S7306, Selleckchem, Houston, TX). After 7 days and 14 days, cells were analyzed for ATP content, mitochondrial membrane potential with tetramethyl rhodamine methyl ester (TMRM), mitochondrial content detected with TOM20 staining and mitochondrial function with the Seahorse Mito Stress test.

### AMPK knockdown using small interfering RNA

Small interfering RNAs (siRNA) against AMPKα1/α2 (sc-45312) and control siRNA (sc-37007) were purchased from Santa Cruz Biotechnology, Inc. On differentiation day 13, cardiac spheres were dissociated into single cells and replated onto Matrigel-coated 12-well plate at 3 × 10^5^ cells per well. After 24 h, cells were transfected with AMPKα1/α2 and control siRNAs using Lipofectamine RNAiMAX (Thermo Fisher Scientific, Waltham, MA). For the assessment of knockdown efficiency, transfected cells were analyzed 72-h post-transfection by qRT-PCR of *PRKAA1* (encoding AMPKα1) and *PRKAA2* (encoding AMPKα2) to determine the optimal dose of siRNAs. Using the optimal dose of siRNAs for AMPK knockdown, transfected cells were collected 7 days post-transfection, and a series of assays were performed to evaluate the changes of ATP, TMRM, TOM20, mtDNA/nDNA and mitochondrial function.

### Pathological modeling

Cardiac spheres were treated with E10 for 7 days to promote metabolic maturation and then treated with pathological stimuli in a low-glucose medium [DMEM-no glucose supplemented with 2-g/L glucose, 10% FBS, 2-mM L-glutamine, 1% penicillin–streptomycin, 0.1-mM non-essential amino acids, 100-µM oleic acid and 50-µM palmitic acid]. For pathological stimulation, cells were treated with 100-µM isoproterenol (I6504, Sigma-Aldrich, St. Louis, MO) in the low-glucose medium in a hypoxic incubator (5% O_2_) at 37 °C for 6 days with the medium refreshed every other day. Changes in pathological phenotype were evaluated with a serial of assays including ATP production, Nile red staining, detection of cell apoptosis, cell viability assay, qRT-PCR and Seahorse assay for glycolysis. Cultures without E10 maturation treatment (immature cells) were used as a control. Cultures treated with pathological stimuli were compared with parallel cultures maintained in low-glucose culture medium in a normoxia incubator (~ 20% O_2_).

### Statistical analysis

The results are presented as the mean ± standard error of the mean (SEM). Data were compared using one-way ANOVA or Student’s t-test (GraphPad) with significant differences defined by *P* < 0.05 (*), *P* < 0.01 (**), *P* < 0.001 (***) and *P* < 0.0001 (****). Sample sizes are given in Figure legends.

## Results

### AMPK activators increased ATP content, mitochondrial membrane potential, mitochondrial content and mtDNA:nDNA ratio in hiPSC-CMs

To examine the role of AMPK activators on the maturation of hiPSC-CMs, we performed a dose screening of AMPK activators on several features of mitochondrial maturation, including ATP content, the ratio of mtDNA:nDNA and mitochondrial membrane potential. The 2D hiPSC-CM cultures (> 90% pure cardiomyocytes) were treated with AMPK activators AMP-mimetric 5-aminoimidazole-4-carboxamide ribonucleoside (AICAR), a thienopyridone derivative (A-769662), a benzimidazole derivative (EX229 or Compound 991) and metformin at various concentrations (see Additional file 1: Results on dose screening of AMPK activators). Compared with DMSO control, all four activators increased the ATP content and mitochondrial membrane potential after 14 days of treatment, and A-769662 and EX229 also increased mtDNA:nDNA ratio in hiPSC-CMs derived from both IMR-90 hiPSCs (Additional file 1: Fig. S1) and SCVI-273 hiPSCs (Additional file 1: Fig. S2). Based on all the three measurements, A-769662 at 100 µM (A100) and 200 µM (A200) and EX229 at 10 µM (E10) and 50 µM (E50) were selected in subsequent studies.

**Fig. 1:**
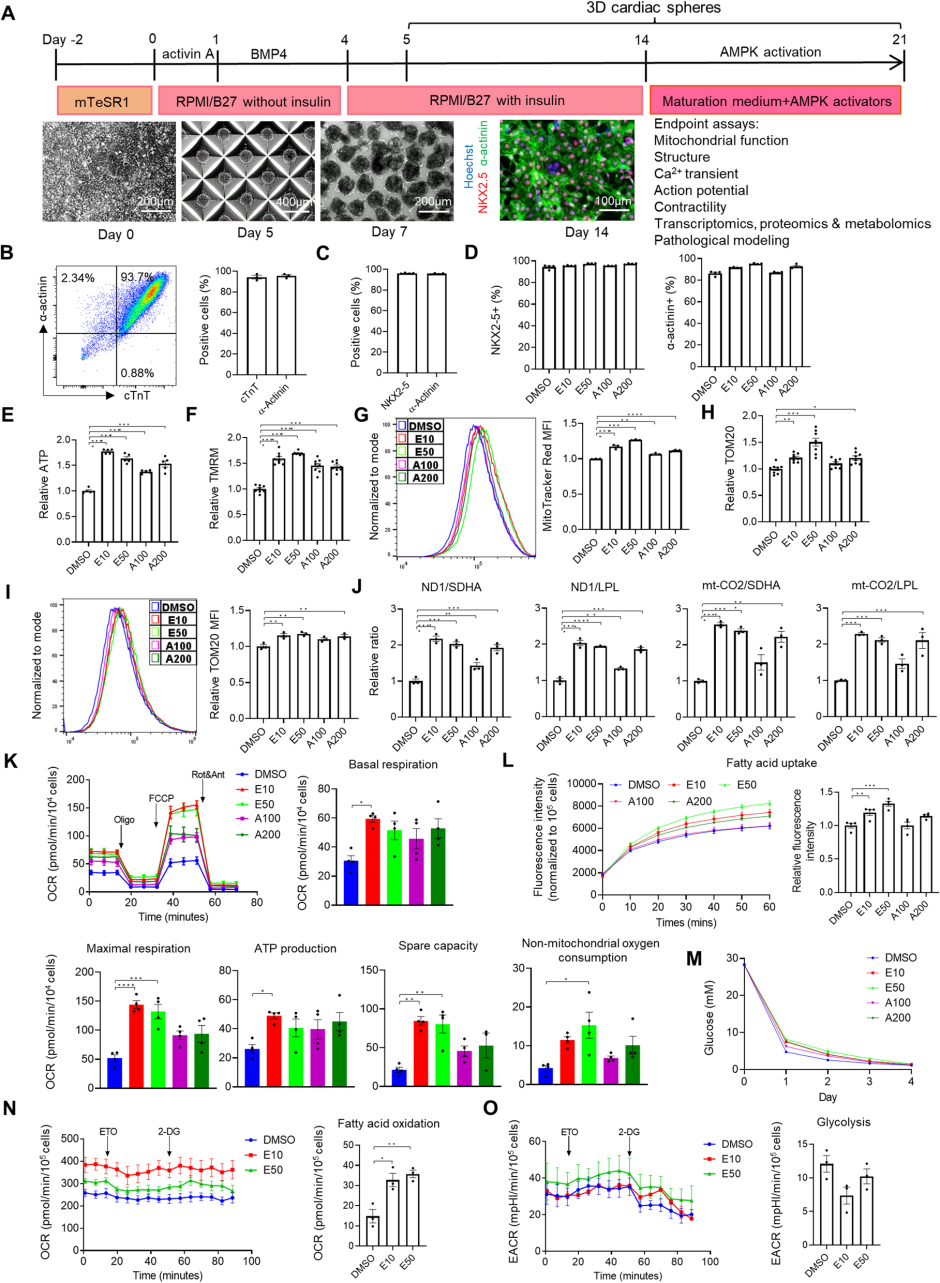
AMPK activators improve metabolic maturation of 3D hiPSC-CMs. **A** Experimental design and cell morphology. Cardiac spheres were generated on day 5 and treated with AMPK activators on day 14 for 7 days before the assessments of cardiomyocyte maturation. **B** Flow cytometry analysis of cTnT and α-actinin on day 14 (*n* = 3 cultures). **C** High-content imaging analysis of NKX2-5 and α-actinin by ArrayScan on day 14 (*n* = 4 cultures). **D** High-content imaging analysis of NKX2-5 and α-actinin by ArrayScan on day 21 (*n* = 4 cultures). **E** ATP content (*n* = 5 cultures). **F** Mitochondrial membrane potentials analyzed by ArrayScan of TMRM (*n* = 8 cultures). **G** Flow cytometry analysis of MitoTracker Red (*n* = 3 cultures). **H** High-content imaging analysis of TOM20 by ArrayScan (*n* = 8 cultures). **I** Flow cytometry analysis of TOM20 (*n* = 3 cultures). **J** Ratios of mtDNA to nDNA (*n* = 3 cultures). **K** Measurement of OCR and quantification of mitochondrial functional parameters including basal respiration, maximal respiration, spare respiratory capacity, non-mitochondrial respiration and ATP production (*n* = 4 cultures). **L** Fatty acid uptake (*n* = 4). **M** Glucose concentration (*n* = 4 cultures). **N** Measurement of OCR and quantification of fatty acid oxidation (the amount of OCR derived from fatty acid oxidation). **O** Measurement of ECAR and quantification of glycolysis (*n* = 4 cultures). Data are presented as mean ± SEM. *P < 0.05; **P < 0.01; ***P < 0.001 and ****P < 0.0001 (one-way ANOVA). 2-DG, 2-Deoxy-d-glucose; A100, A-769662 at 100 µM; A200, A-769662 at 200 µM; AA, Activin A, BMP4, Bone morphogenetic protein 4; cTnT, Cardiac troponin T; DMSO, Dimethyl sulfoxide; E10, EX229 at 10 µM; E50, EX229 at 50 µM; ECAR, Extracellular acidification rate; ETO, Etomoxir; FCCP, Carbonyl cyanide p-(trifluoromethoxy) phenylhydrazone; LPL, Lipoprotein lipase; MFI, Mean fluorescence intensity; mt-CO2, Mitochondrially encoded cytochrome c oxidase II; mtDNA, Mitochondrial DNA; ND1, Mitochondrially encoded NADH dehydrogenase 1; nDNA, Nuclear DNA; NKX2-5, NK2 homeobox 5; OCR, Oxygen consumption rate; Oligo, Oligomycin; Rot/Ant, Rotenone/antimycin A; SDHA, Succinate dehydrogenase complex flavoprotein subunit A and TMRM, Tetramethylrhodamine, methyl ester

We further examined the effect of AMPK activators on mitochondria maturation in 3D culture of hiPSC-CMs. Cardiac spheres were generated by microscale tissue engineering from hiPSC differentiation cultures [12, 13]. The maturation outcomes were then measured after cardiac spheres were treated with A-769662 or EX229 for 7 or 14 days starting on differentiation day 14 (Fig. 1A). The 3D cultures contained highly enriched hiPSC-CMs (> 90% pure cardiomyocytes; Additional file 2: Video S1) as analyzed by flow cytometry (Fig. 1B) and high-content imaging (Fig. 1C). The hiPSC-CM purity of the cultures remained similar after these cells were treated with A-769662 or EX229 (Fig. 1B–D, detailed in Additional file 1: Results). At treatment day 7, both EX229 and A-769662 significantly increased the ATP content (Fig. 1E). Cells treated with E10 had the highest ATP content levels among all tested conditions (> 1.7-fold higher in E10-treated cells than that in DMSO-treated cells) (Fig. 1E). At treatment day 14, the effect of elevated ATP content with treatment at optimal doses showed the same trend as at treatment day 7 (Additional file 1: Fig. S3B).

We also investigated the effect of these two AMPK activators on the mitochondrial membrane potential using TMRM, a cell-permeant and cationic dye that accumulates in active mitochondria with intact membrane potentials. At treatment day 7, cells in all four tested conditions showed increased mean fluorescence intensity of TMRM compared with DMSO-treated cells (Fig. 1F). In agreement with the effect on ATP content, EX229 was more potent in promoting mitochondrial membrane potential than A-769662. Similar increased TMRM levels were also observed in 3D hiPSC-CMs at treatment day 14 (Additional file 1: Fig. S3C). E10, E50 and A200 also increased mean fluorescence intensity of MitoTracker Red, another indicator of mitochondrial membrane potential at treatment day 7 (Fig. 1G) and day 14 (Additional file 1: Fig. S3D).

In addition, we examined the expression of TOM20, an indicator of mitochondrial content, in 3D hiPSC-CMs treated with A-769662 and EX229. Both quantitative methods of ArrayScan and flow cytometry detected elevated expression of TOM20 in E10-, E50- and A200-treated cultures (Fig. 1H and I; Additional file 1: Fig. S3E and F).

We further evaluated mitochondrial DNA development by measuring mtDNA:nDNA ratio in 3D hiPSC-CMs treated with A-769662 and EX229 (Fig. 1J; Additional file 1: Fig. S3G). Compared with DMSO treatment, all other treatments resulted in the increased mtDNA:nDNA ratio of ND1/SDHA and ND1/LPL. Higher levels of increase were observed in cells treated with E10, E50 and A200 than with A100. E10, E50 and A200 also increased the mtDNA:nDNA ratio of mt-CO2/SDHA and mt-CO2/LPL, and E10-treated cells had the highest mtDNA:nDNA ratio among conditions, reaching a more than two-fold increase in mtDNA:nDNA ratio compared with that of DMSO-treated cells.

These results showed that AMPK activators increased ATP content, mitochondrial membrane potential and mtDNA:nDNA ratio in hiPSC-CMs, promoting mitochondrial development.

### AMPK activators promoted mitochondrial function and FAO

To characterize mitochondrial function, we measured major aspects of mitochondrial coupling and respiratory control—basal respiration, ATP production, maximal respiration, spare respiratory capacity and non-mitochondrial respiration—by the sequential additions of oligomycin (ATP synthase inhibitor), FCCP (an uncoupler of oxidative phosphorylation) and rotenone and antimycin A (electron inhibitors) using the Seahorse Extracellular Flux Analyzer. At treatment day 7, AMPK activator-treated cells had higher basal, maximal respiration, spare respiratory capacity, non-mitochondrial respiration and ATP production than DMSO-treated cells (Fig. 1K). Among the treatment conditions, E10 resulted in the highest basal, maximal respiration, ATP production, spare respiratory capacity and non-mitochondrial respiration (Fig. 1K). At treatment day 14, the effect of E10 on basal, maximal respiration, ATP production and non-mitochondrial respiration remained similar to treatment day 7 (Additional file 1: Fig. S3H).

We next examined if enhanced mitochondrial function in AMPK activator-treated cells coincided with the metabolic changes in the usage of energetic substrates. Compared with DMSO-treated cells, EX229-treated hiPSC-CMs had increased fatty acid uptake as detected by a fluorometric assay kit, and EX229 was more potent in enhancing the fatty acid uptake than A-769662 (Fig. 1L; Additional file 1: Fig. S3I). The consumption of glucose did not significantly change among conditions during monitoring for 4 days after the treatment (Fig. 1M). These observations indicate that treatment with EX229 boosted the usage of fatty acid.

Since EX229 was more potent than A-769662 in promoting mitochondrial function and enhancing the fatty acid uptake, we also examined the level of FAO and glycolysis in EX229-treated hiPSC-CMs. By monitoring OCR using the Seahorse XF24 Extracellular Flux Analyzer, which is an established assay for FAO measurement [3], cells were treated with ETO, a specific inhibitor of CPT1 which is an enzyme that mediates internalization of fatty acids into the mitochondrial matrix for oxidation, and the level of FAO was then calculated by the ETO-induced reduction in OCR normalized to the number of cells. Compared with DMSO-treated cells, EX229-treated hiPSC-CMs had higher levels of basal respiration and FAO (Fig. 1N). The level of glycolysis in EX229-treated hiPSC-CMs was not significantly different from that in the DMSO-treated cells (Fig. 1O). Therefore, AMPK activator EX229 promoted mitochondrial function and FAO.

### AMPK inhibitor reduced mitochondrial maturation and abolished the effect of AMPK activators on mitochondrial maturation

**Fig. 2:**
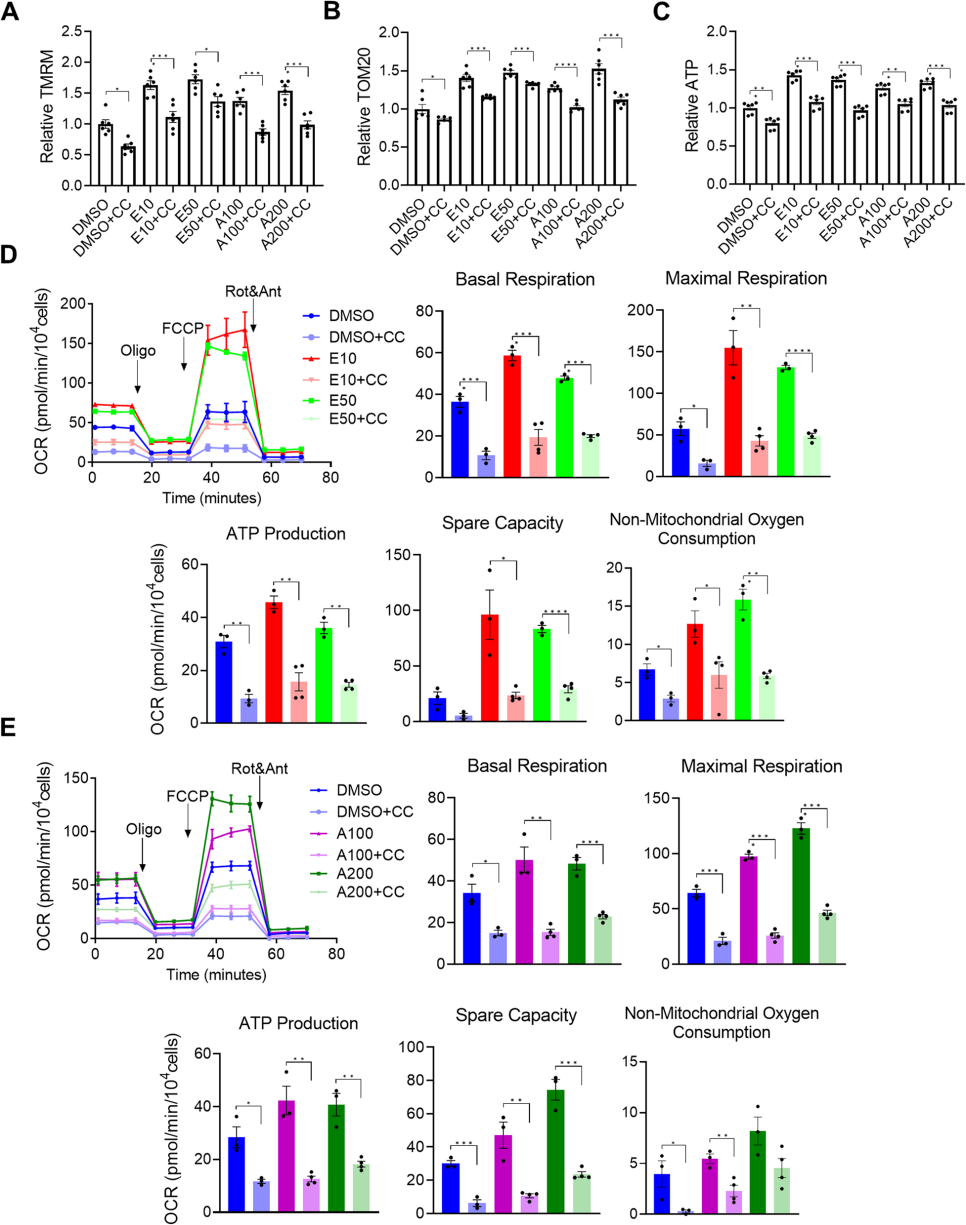
AMPK inhibition using Compound C inhibits the metabolic maturation of hiPSC-CMs. Measurements of relative **A** ATP content, **B** TMRM and **C** TOM20. **D**, **E** Measurement of OCR and quantification of basal respiration, maximal respiration, ATP production, spare respiratory capacity and non-mitochondrial respiration. The effect of EX229 (**D**) and A-769662 (**E**) on mitochondrial function was abolished with Compound C. All measurements were normalized to cell counts (*n* = 4 cultures). Data are presented as mean ± SEM. *P < 0.05; **P < 0.01; ***P < 0.001 and ****P < 0.0001 (one-way ANOVA). A100, A-769662 at 100 µM; A200, A-769662 at 200 µM; DMSO, Dimethyl sulfoxide; E10, EX229 at 10 µM; E50, EX229 at 50 µM; FCCP, Carbonyl cyanide p-(trifluoromethoxy) phenylhydrazone; OCR, Oxygen consumption rate; Oligo, Oligomycin; Rot/Ant, Rotenone/antimycin A and TMRM, Tetramethylrhodamine, methyl ester

To further evaluate the role of AMPK in mitochondrial maturation of hiPSC-CMs, we examined the effect of a specific chemical inhibitor of AMPK, Compound C, on mitochondrial membrane potential, mitochondrial content and ATP content in 3D hiPSC-CMs. Following 7 days of treatment with Compound C, levels of mitochondrial membrane potential (Fig. 2A), mitochondrial content (Fig. 2B) and ATP content (Fig. 2C) in 3D hiPSC-CMs were reduced compared with cultures treated with DMSO control. In addition, the levels of mitochondrial membrane potential (Fig. 2A), mitochondrial content (Fig. 2B) and ATP content (Fig. 2C) in 3D hiPSC-CMs co-treated with an AMPK activator and Compound C were lower than in cultures treated with AMPK activator alone (comparing E10 + CC vs. E10, E50 + CC vs. E50, A100 + CC vs. A100 and A200 + CC vs. A200).

**Fig. 3:**
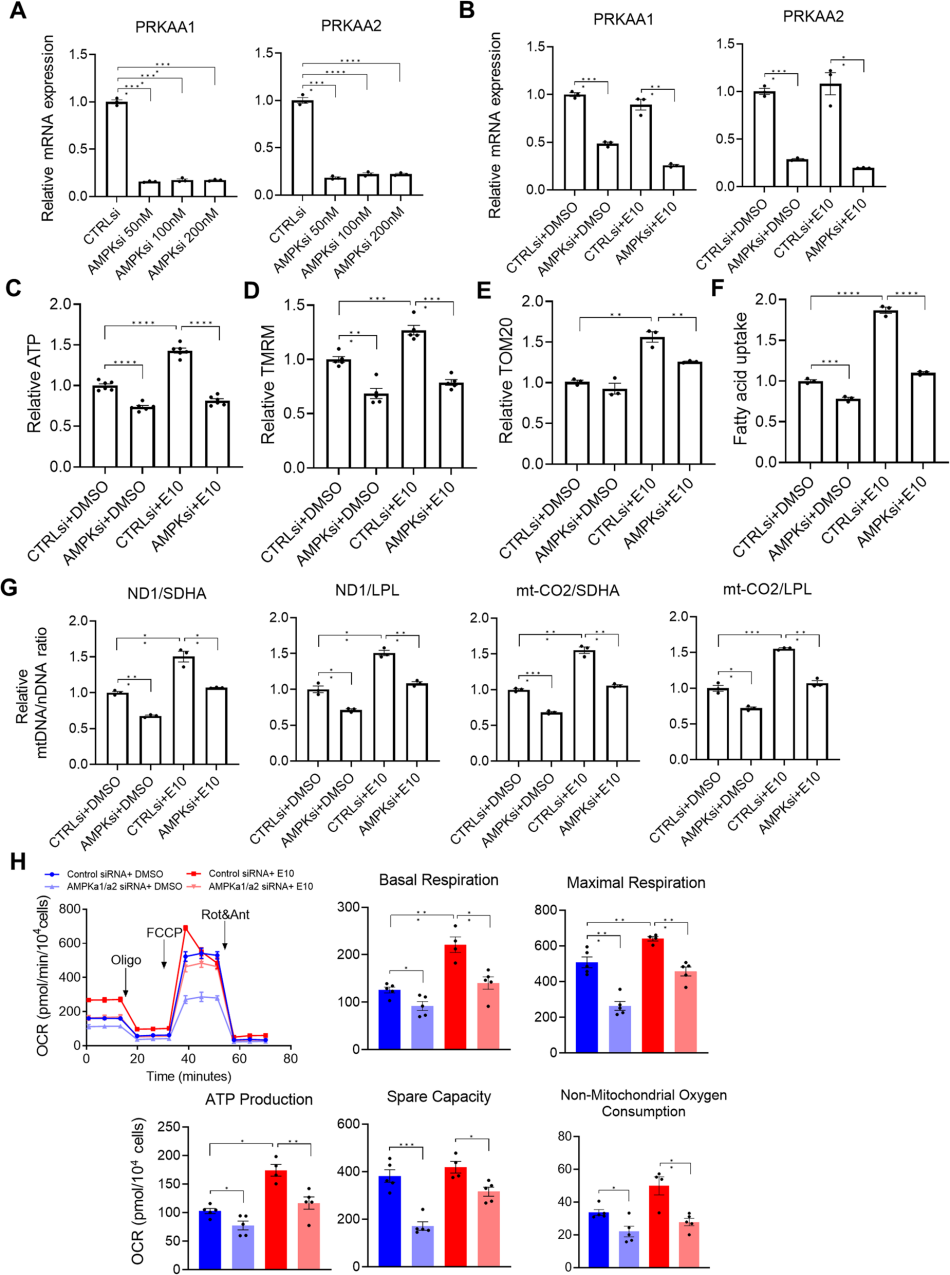
siRNA knockdown of AMPK inhibits the metabolic maturation of hiPSC-CMs. **A** Relative mRNA levels of PRKAA1 and PRKAA2 in hiPSC-CMs transfected with siRNA at doses 50, 100 and 200 μM compared with control siRNA after 72 h (*n* = 3 cultures). **B** Relative mRNA levels of PRKAA1 and PRKAA2 in hiPSC-CMs transfected with siRNA at 50 μM compared with control siRNA after 7 days (*n* = 3 cultures). Measurements of relative **C** ATP content (*n* = 5), **D** TMRM (*n* = 6 cultures), **E** TOM20 (*n* = 4 cultures) and **F** fatty acid uptake capacity (*n* = 3 cultures) of AMPK siRNA transfected hiPSC-CMs treated with DMSO or E10 compared with control siRNA transfected hiPSC-CMs treated with DMSO or E10. (H) Relative ratio of mtDNA and nDNA. **G** Mitochondrial function measured in DMSO- or E10-treated hiPSC-CMs transfected with control or AMPK siRNA. All measurements were normalized to cell counts (*n* = 4 or 5 cultures). Data are presented as mean ± SEM. *P < 0.05; **P < 0.01; ***P < 0.001 and ****P < 0.0001 (one-way ANOVA). DMSO, Dimethyl sulfoxide; E10, EX229 at 10 µM; FCCP, Carbonyl cyanide p-(trifluoromethoxy) phenylhydrazone; LPL, Lipoprotein lipase; mt-CO2, Mitochondrially encoded cytochrome c oxidase II; mtDNA, Mitochondrial DNA; ND1, Mitochondrially encoded NADH dehydrogenase 1; nDNA, Nuclear DNA; OCR, Oxygen consumption rate; Oligo, Oligomycin; PRKAA1, Protein kinase AMP-activated catalytic subunit alpha 1; PRKAA2, Protein kinase AMP-activated catalytic subunit alpha 2; Rot/Ant, Rotenone/antimycin A; SDHA, Succinate dehydrogenase complex flavoprotein subunit A; siRNA, Small interfering RNA and TMRM, Tetramethylrhodamine, methyl ester

We further examined if Compound C affected mitochondrial function by the Seahorse Mito Stress assay. Following 7 days of Compound C treatment of 3D hiPSC-CMs (derived from IMR90 hiPSCs), the levels of basal respiration, maximal respiration, ATP production, spare respiratory capacity and non-mitochondrial oxygen consumption were lower than in cultures treated with DMSO control (Fig. 2D and E). In addition, Compound C treatment abolished increased mitochondrial function in AMPK activator-treated cultures (Fig. 3C, D). After 14 days of the treatment, Compound C also reduced ATP content (Additional file 1: Fig. S4A), mitochondrial membrane potential (Additional file 1: Fig. S4B), mitochondrial content (Additional file 1: Fig. S4C) and mitochondrial function (Additional file 1: Fig. S4D and E).

In addition, we also examined the effect of Compound C in hiPSC-CMs derived from another cell line. The negative effect of Compound C on ATP content, mitochondrial membrane potential, mitochondrial content and mitochondrial function was also observed in 3D hiPSC-CMs derived from SCVI273 hiPSCs at treatment day 7 (Additional file 1: Fig. S5) and day 14 (Additional file 1: Fig. S6).

Taken together, these results indicate that AMPK inhibitor Compound C reduced ATP content, mitochondrial content, mitochondrial membrane potential and mitochondrial function in 3D hiPSC-CMs derived from both IMR90 hiPSCs and SCVI273 hiPSCs, supporting a role of AMPK in mitochondrial maturation of hiPSC-CMs.

### AMPK knockdown using small interfering RNA inhibited mitochondrial maturation

We next investigated the role of AMPK in mitochondrial maturation by genetic knockdown of *AMPK*, which was achieved by the expression of two sets of small interfering RNAs (siRNAs) that targeted *PRKAA1* (encoding AMPKα1)* a*nd *PRKAA2* (encoding AMPKα2). hiPSC-CMs derived from IMR90 hiPSCs were transfected with *AMPK* siRNAs or a scrambled control siRNA followed by E10 treatment for 7 days. Compared with the control siRNA, *AMPK* siRNAs caused efficient knockdown of *PRKAA1 a*nd *PRKAA2* expression at 50–200 nM (Fig. 3A). In hiPSC-CMs treated with AMPK activator E10 or DMSO, levels of *PRKAA1 a*nd *PRKAA2* expression remained lower in AMPK siRNAs cultures than those in the control siRNA cultures after siRNA treatment for 7 days (Fig. 3B).

We then examined the effect of *AMPK* knockdown on mitochondrial maturation in cultures treated with E10 or DMSO. Levels of ATP content, TMRM and TOM20 in cultures treated with *AMPK* siRNAs were significantly lower than those in the control siRNA cultures (Fig. 3C–E), indicating knockdown of *AMPK* reduced ATP content, mitochondrial membrane potential and mitochondrial content. Compared with the control siRNA cultures, *AMPK* siRNAs cultures also had decreased fatty acid uptake (Fig. 3F) and decreased mtDNA/nDNA ratio (Fig. 3G). In addition, *AMPK* knockdown abolished the positive effect of E10 on ATP content, mitochondrial membrane potential, mitochondrial content, mtDNA/nDNA ratio and fatty acid uptake (Fig. 3C–G).

We further examined the outcomes of *AMPK* knockdown on mitochondrial function using Seahorse Mito Stress assay. *AMPK* siRNAs cultures had significantly lower levels of basal respiration, maximal respiration, ATP production, spare respiratory capacity and non-mitochondrial oxygen consumption than did the control siRNA cultures (Fig. 3H). *AMPK* knockdown also abolished the positive effect of E10 on these parameters associated with mitochondrial function (Fig. 3H).

Therefore, the results from both pharmacological and genetic inhibition of AMPK consistently support a functional role of AMPK in mitochondrial maturation of hiPSC-CMs.

### AMPK activators promoted structural maturation, calcium handling, electrophysiology and contractility index

**Fig. 4:**
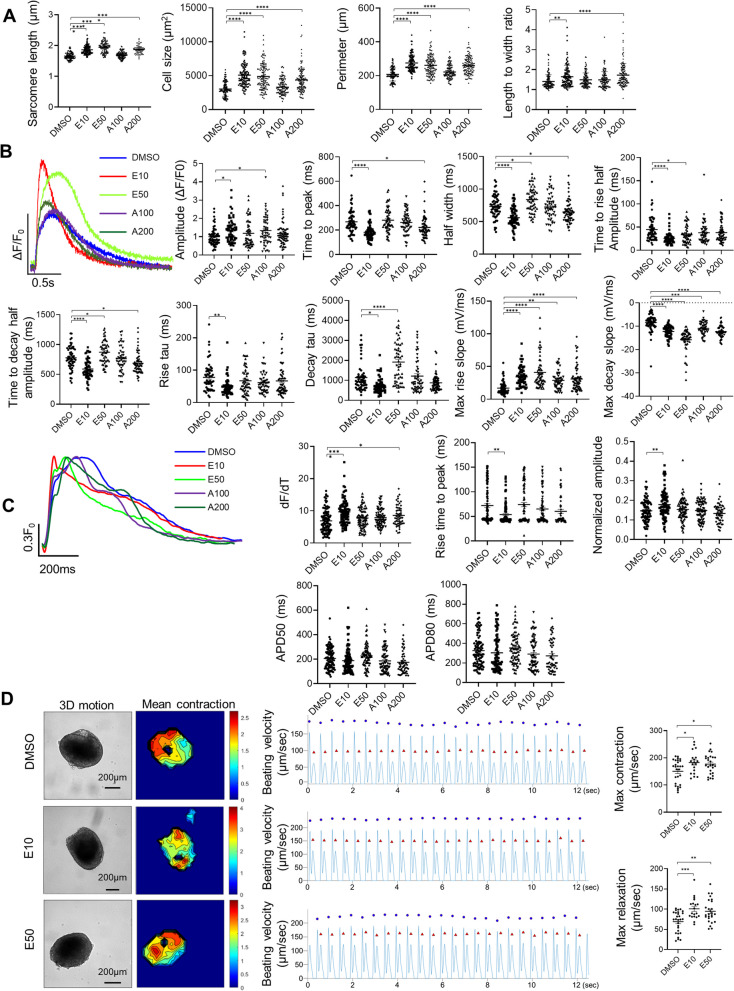
AMPK activation improves structural and functional maturation of hiPSC-CMs. **A** Structural analysis of sarcomere length, cell size, perimeter and length to width ratio. *n* = 105–112 cells. **B** Representative Ca^2+^ transient traces from DMSO- and AMPK activator-treated hiPSC-CMs and quantification of Ca^2+^ transient characteristics (*n* = 52–70 cells). **C** Representative action potential traces and quantification of dF/dT, time to peak, normalized amplitude, AP50 and AP80 (*n* = 55–95 cells). **D** Representative image and heatmap (left) depicting time-averaged magnitude of all motion and tracing (middle) of average beating speed followed by the analysis of contraction velocity and relaxation velocity of 3D hiPSC-CMs (*n* = 19–28 spheres). Beating activities were recorded at 0.5 Hz. Data are presented as mean ± SEM. *P < 0.05; **P < 0.01; ***P < 0.001 and ****P < 0.0001 (one-way ANOVA). 3D, Three-dimensional; A100, A-769662 at 100 µM; A200, A-769662 at 200 µM; AP50, Action potential duration measured at 50%; AP80, Action potential duration measured at 80%; DMSO, Dimethyl sulfoxide; E10, EX229 at 10 µM and E50, EX229 at 50 µM

To evaluate structural maturation, we performed immunostaining of α-actinin, a major component of the Z-line, on hiPSC-CMs treated with EX229 and A-769662 for 7 days and quantified sarcomere length, cell area, cell perimeter and length/width ratio (Additional file 1: Fig. S7 and Fig. 4A). Compared with sarcomere length of DMSO-treated cells (1.658 ± 0.011 µm), sarcomere length significantly increased in E10- (1.861 ± 0.013 µm), E50- (1.949 ± 0.017 µm) and A200- (1.868 ± 0.016 µm) treated hiPSC-CMs (Fig. 4A). In addition, DMSO-treated cells were smaller (3.037 ± 103 µm^2^) than those treated with E10 (5.096 ± 164 µm^2^), E50 (4.892 ± 173 µm^2^) and A200 (4.385 ± 153 µm^2^) based on cell area (Fig. 4A). Similarly, E10, E50 and A200 treatment increased cell perimeters (Fig. 4A). E10 and A200 also increased length/width ratio of the cells (Fig. 4A).

We next investigated the calcium handling properties of hiPSC-CMs using line-scan confocal imaging after cells were loaded with the intracellular calcium dye, Fluo-4AM. E10- and A100-treated cells displayed significantly higher calcium transient amplitude (Fig. 4B). All treatments with AMPK activators led to significantly higher maximal rise slope and maximal decay slope than DMSO control (Fig. 4B). Time to rise half amplitude was significantly shorter with E10 and E50 treatments. E10- and A200-treated cells had significantly shortened the time to peak amplitude and the time to decay half amplitude. Notably, only E10-treated cells had decreased rise tau and decay tau, making E10 the most potent treatment with enhanced calcium transient kinetics based on all parameters examined (Fig. 4B). In addition, enhanced calcium transient kinetics were also observed in E10-treated hiPSC-CMs after 14 days of the treatment (Additional file 1: Fig. S3K). Together, these results indicate that AMPK activators significantly increased the kinetics of calcium transients, a functional characteristic of more mature hiPSC-CMs.

We further investigated the electrophysiological properties of hiPSC-CMs using FluoVolt probe and confocal imaging. E10-treated hiPSC-CMs had significantly higher peak amplitude and shorter peak rise time. The upstroke velocity was faster in both E10- and A200-treated cells while the action potentials APD50 and APD80 did not change (Fig. 4C). In addition, compared with DMSO-treated cells, E10- and E50-treated 3D hiPSC-CMs had higher average maximum contraction (DMSO, 150.37 ± 8.18 µm/s; E10, 182.78 ± 9.33 µm/s and E50, 177.21 ± 7.20 µm/s) and relaxation (DMSO, 69.03 ± 4.92 µm/s; E10, 100.90 ± 6.11 µm/s and E50, 94.83 ± 5.63 µm/s) velocities (Fig. 4D).

Together, these results indicate that treatment of AMPK activators promoted the functional maturation of hiPSC-CMs.

### AMPK activator-treated 3D hiPSC-CMs had increased expression of genes involved in controlling mitochondrial properties and cardiac structural and functional features

**Fig. 5:**
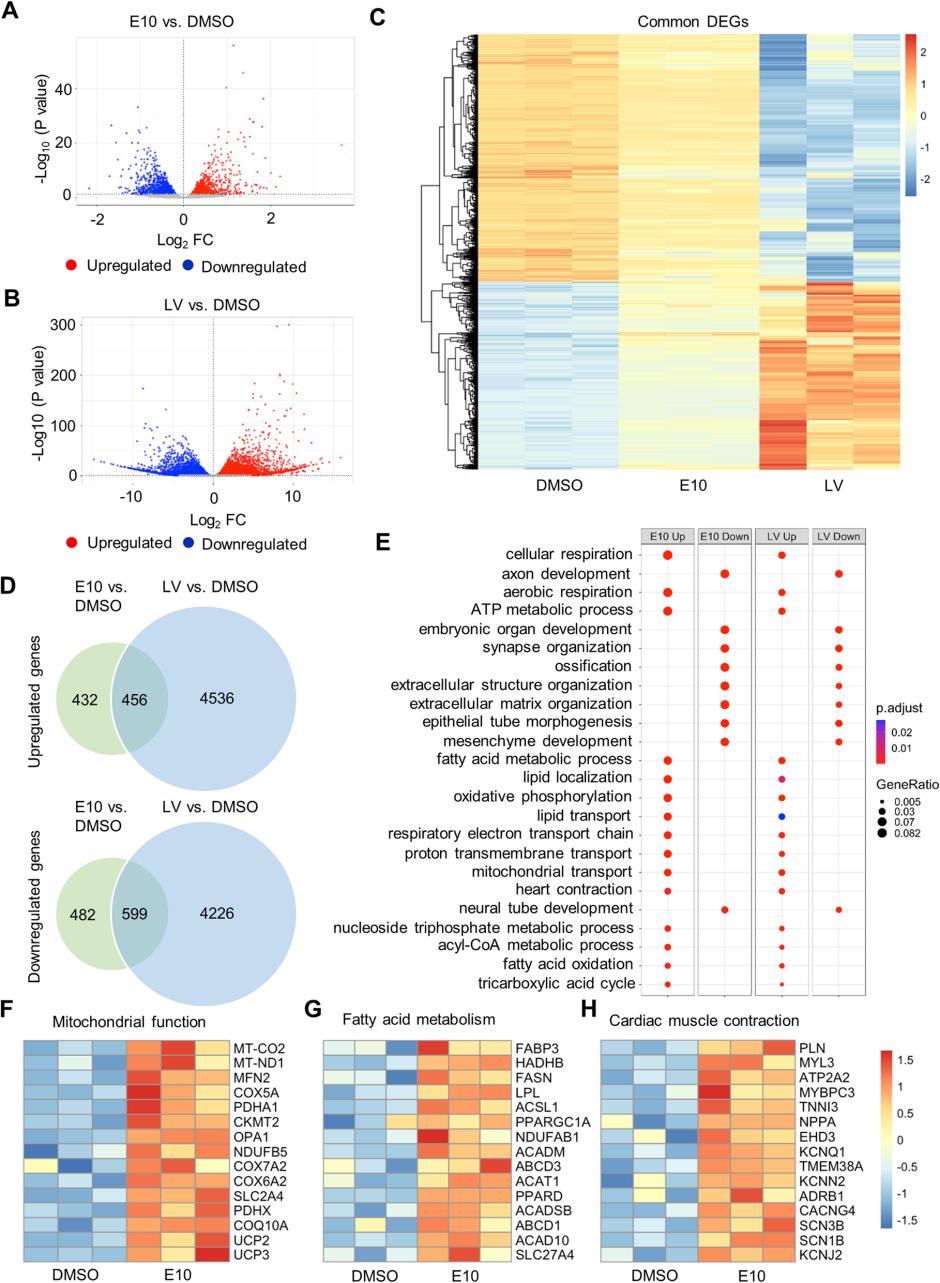
AMPK activation alters the gene expression profile in hiPSC-CMs identified by RNA-seq. Volcano plots portray log_2_ (fold change) vs. negative log_10_(adjusted P value) for differentially expressed genes (DEGs) in **A** E10-treated hiPSC-CMs and **B** tissues from human left ventricle (LV) compared with DMSO control (*n* = 3 cultures or tissue samples). **C** Heatmap showing common DEGs in E10-treated hiPSC-CMs and LV compared with DMSO-treated hiPSC-CMs. **D** Venn diagram showing the number of upregulated and downregulated DEGs in E10-treated hiPSC-CMs versus DMSO-treated hiPSC-CMs and LV versus DMSO-treated hiPSC-CMs. **E** Upregulated and downregulated GO terms in E10-treated hiPSC-CMs and LV compared with DMSO-treated hiPSC-CMs. Heatmaps of DEGs in E10-treated hiPSC-CMs vs. DMSO-treated hiPSC-CMs associated with **F** mitochondrial function, **G** fatty acid metabolism and **H** cardiac muscle contraction. E10, EX229 at 10 µM; LV, Heart tissue samples from pediatric left ventricle and RNA-seq, RNA-sequencing

We next investigated the effect of AMPK activator treatment on gene expression profile (we chose E10 for the experiments given it showed most potent effect on maturation phenotypes). RNA-sequencing (RNA-seq) analysis was performed on 3D hiPSC-CMs treated with E10 or DMSO for 7 days as well as three heart tissue samples from pediatric left ventricle (LV). At the threshold of adjusted *P* value < 0.05, we identified 1969 differentially expressed genes (DEGs) in E10-treated cells compared with DMSO-treated cells, including 888 upregulated genes and 1081 downregulated genes (Fig. 5A). DEGs (9817) were identified in LV samples compared with DMSO-treated cells, including 4992 upregulated genes and 4825 downregulated genes (Fig. 5B). Common DEGs were visualized in a heatmap showing a differential gene expression pattern across the three groups (Fig. 5C). Specifically, there were 1055 commonly expressed DEGs between E10-treated cells and LV compared with DMSO-treated cells, including 456 upregulated and 599 downregulated DEGs (Fig. 5D). Top commonly expressed DEGs between E10-treated hiPSC-CMs and LV were listed in Additional file 1: Table S3.

We performed GO analysis on the DEGs in E10- vs. DMSO-treated hiPSC-CMs and LV vs. DMSO-treated hiPSC-CMs. Strikingly, a large number of biological processes were commonly enhanced in E10-treated cells and LV. Most top upregulated GO terms represented mitochondrial metabolism including cellular respiration, aerobic respiration, oxidative phosphorylation, ATP metabolic process, respiratory electron transport chain, nucleoside triphosphate metabolic process, proton transmembrane transport, acyl-CoA metabolic process, tricarboxylic acid cycle and mitochondrial transport (Fig. 5E). These upregulated GO terms also included fatty acid metabolic process, lipid localization, FAO, lipid transport and heart contraction, representing the upregulation of fatty acid metabolism, cardiac structural and functional development in E10-treated cells (Fig. 5E). In addition, E10 downregulated GO terms associated with extracellular matrix organization, extracellular structure organization, skeletal system development, mesenchyme development, embryonic organ development and neural tube development (Fig. 5E).

The investigation of individual gene changes revealed a number of DEGs that are critical for mitochondrial function in E10-treated hiPSC-CMs (Fig. 5F). These included *MT-ND1* (mitochondrially encoded NADH dehydrogenase 1), *MT-CO2* (mitochondrially encoded cytochrome c oxidase II), *COQ10A* (coenzyme Q10A), *CKMT2* (creatine kinase, mitochondrial 2), *OPA1* (OPA1 mitochondrial dynamin-like GTPase), *MFN2* (mitofusin 2), *UCP2* (uncoupling protein 2), *UCP3* (uncoupling protein 3), *NDUFB5* (NADH:ubiquinone oxidoreductase subunit B5), *SLC2A4* (solute carrier family 2 member 4), *PDHA1* (pyruvate dehydrogenase E1 subunit alpha 1), *PDHX* (pyruvate dehydrogenase complex component X) and COX (cytochrome c oxidase) subunits *COX5A*, *COX6A2* and *COX7A2*. The upregulation of *MT-ND1* and *MT-CO2* was consistent with the result in mtDNA/nDNA ratio. The increased expression of *SLC2A4* coincided with the theory that the switch of glucose transporter from *SLC2A1* to *SLC2A4* occurs along with the CM maturation. PDH complex consists of key enzymes that link the glycolysis metabolic pathway to the tricarboxylic acid (TCA) cycle and catalyze the process of pyruvate decarboxylation by converting pyruvate to acetyl-coenzyme A and carbon dioxide, where *PDHA1* plays a key role in maintaining the function of PDH complex. *DLAT* (dihydrolipoamide S-acetyltransferase) and *DLD* (dihydrolipoamide dehydrogenase) which encode the other two subunits of PDH were also upregulated, suggesting the enhanced capacity of pyruvate decarboxylation. We also found multiple DEGs that encode the key enzymes in TCA cycle, including *CS* (citrate synthase), *ACO2* (aconitase 2), *IDH3A* (isocitrate dehydrogenase (NAD( +)) 3 catalytic subunit alpha), *OGDH* (oxoglutarate dehydrogenase), *DLST* (dihydrolipoamide S-succinyltransferase), *DLD*, *SDHB* (succinate dehydrogenase complex iron sulfur subunit B), *SDHD* (succinate dehydrogenase complex subunit D), *FH* (fumarate hydratase) and the cytosolic isozyme *MDH1* (malate dehydrogenase 1). In complex I of the respiratory chain, in addition to *NDUFB5*, some other subunits of NADH:ubiquinone oxidoreductase were also upregulated, including *NDUFA4*, *NDUFA5*, *NDUFA10*, *NDUFAB1*, *NDUFAF4*, *NDUFB3*, *NDUFB9*, *NDUFS1*, *NDUFS2* and *NDUFS3*. *SDHB* and *SDHD* in complex II together with ubiquinol-cytochrome c oxidoreductase complex (UQCR) subunits *UQCR10*, *UQCRB*, *UQCRC1*, *UQCRC2, UQCRFS1* and *UQCRH* in complex III of the respiratory chain were upregulated. Additionally, we also observed the upregulation of some other COX subunits in complex IV including *COX4I1*, *COX6B1*, *COX6C*, *COX7B*, *COX7C* and the COX assembly subunit *COX14*. Genes that encode ATP synthase, for example, *ATP5F1A* (*ATP5A1*, ATP synthase F1 subunit alpha), *ATP5F1B* (*ATP5B*, ATP synthase F1 subunit beta) and *ATP5F1C* (*ATP5C*, ATP synthase F1 subunit gamma), were also upregulated. Our mitochondrial function test on E10-treated cells using Seahorse showed significant increased proton leak, which is known to be activated by enhanced level of oxidative phosphorylation [14]. UCP2 and UCP3 together with another upregulated gene, SOD2 (superoxide dismutase 2), enhanced their expression to mediate the potential oxidative stress induced by the increased proton leak [14–17].

Several key genes involved in fatty acid metabolism were upregulated in E10-treated cells (Fig. 5G). *FABP3* (fatty acid-binding protein 3) is a critical gene in transporting long-chain fatty acids from cytoplasm to mitochondria. *SLC27A4*, also known as *FATP4*, is a fatty acid transport protein that functions in translocation of long-chain fatty acids cross the plasma membrane. *PPARGC1A* (peroxisome proliferator-activated receptor gamma coactivator 1-alpha), also known as *PGC-1a* (PPARG coactivator 1 alpha), is a master regulator of mitochondrial biogenesis. *ACSL1* (acyl-CoA synthetase long-chain family member 1) plays a key role in lipid biosynthesis and fatty acid degradation. Other key upregulated genes that participated in FAO were upregulated in E10-treated cells, included *HADHB* (hydroxyacyl-CoA dehydrogenase trifunctional multienzyme complex subunit beta), *FASN* (fatty acid synthase), the enzymes of acyl-CoA dehydrogenase family (ACADs) *ACAD10*, *ACADM*, *ACADSB*, *ACAT1* (acetyl-CoA acetyltransferase 1) and ATP-binding cassette (ABC) transporters *ABCA5*, *ABCB4*, *ABCC5*, *ABCC9*, *ABCD1*, *ABCD3*, *ABCG1* and *TAP1*.

Additionally, E10-treated cells had upregulated *PPARD* (peroxisome proliferator-activated receptor delta), which was recently identified for its role in inducing metabolic and contractile maturation of hiPSC-CMs [18]. E10-treated cells also had upregulated *MB* (myoglobin) which transfers oxygen from the cell membrane to the mitochondria, indicating increased cellular respiration in mitochondria. These results were consistent with improved mitochondrial function in E10-treated hiPSC-CMs.

E10 treatment also upregulated genes that play an important role in the maturation of electrophysiology and Ca^2+^ handling of cardiomyocytes (Fig. 5H). These genes included *KCNJ2* (potassium inwardly rectifying channel subfamily J member 2), *PLN* (phospholamban), *ATP2A2* (ATPase sarcoplasmic/endoplasmic reticulum Ca^2+^ transporting 2), *TNNI3* (troponin I3, cardiac type)*, NPPA* (natriuretic peptide A), *ADRB1* (adrenoceptor beta 1), *KCNN2* (potassium calcium-activated channel subfamily N member 2), *KCNQ1* (potassium voltage-gated channel subfamily Q member 1), *SCN1B* (sodium voltage-gated channel beta subunit 1), *SCN3B* (sodium voltage-gated channel beta subunit 3), *CACNG4* (calcium voltage-gated channel auxiliary subunit gamma 4), *MYL3* (myosin light chain 3), *MYBPC3* (myosin-binding protein C3), *TMEM38A* (transmembrane protein 38A) and *EHD3* (EH domain containing 3). In addition, we observed the decreased expression of the automaticity ion channel *HCN4* (hyperpolarization-activated cyclic nucleotide-gated potassium channel 4). Notably, many of these upregulated genes were reported as cardiomyocyte maturation markers. For example, *TNNI3* represents one of the outstanding cardiomyocyte maturation markers [19]. In addition, majority of these upregulated genes in E10-treated cells were also upregulated in LV samples compared with DMSO-treated cells, although the differences in expression levels of some genes were also noted (Additional file 1: Fig. S8).

Together, these observations—increased expression of genes involved in controlling mitochondrial properties, cardiac structure and cardiac function—show that E10-treated hiPSC-CMs had an improved molecular signature indicative of more mature cardiomyocytes.

### Proteomics analysis revealed upregulation of key regulators on mitochondrial function and fatty acid metabolism in E10-treated 3D hiPSC-CMs

**Fig. 6:**
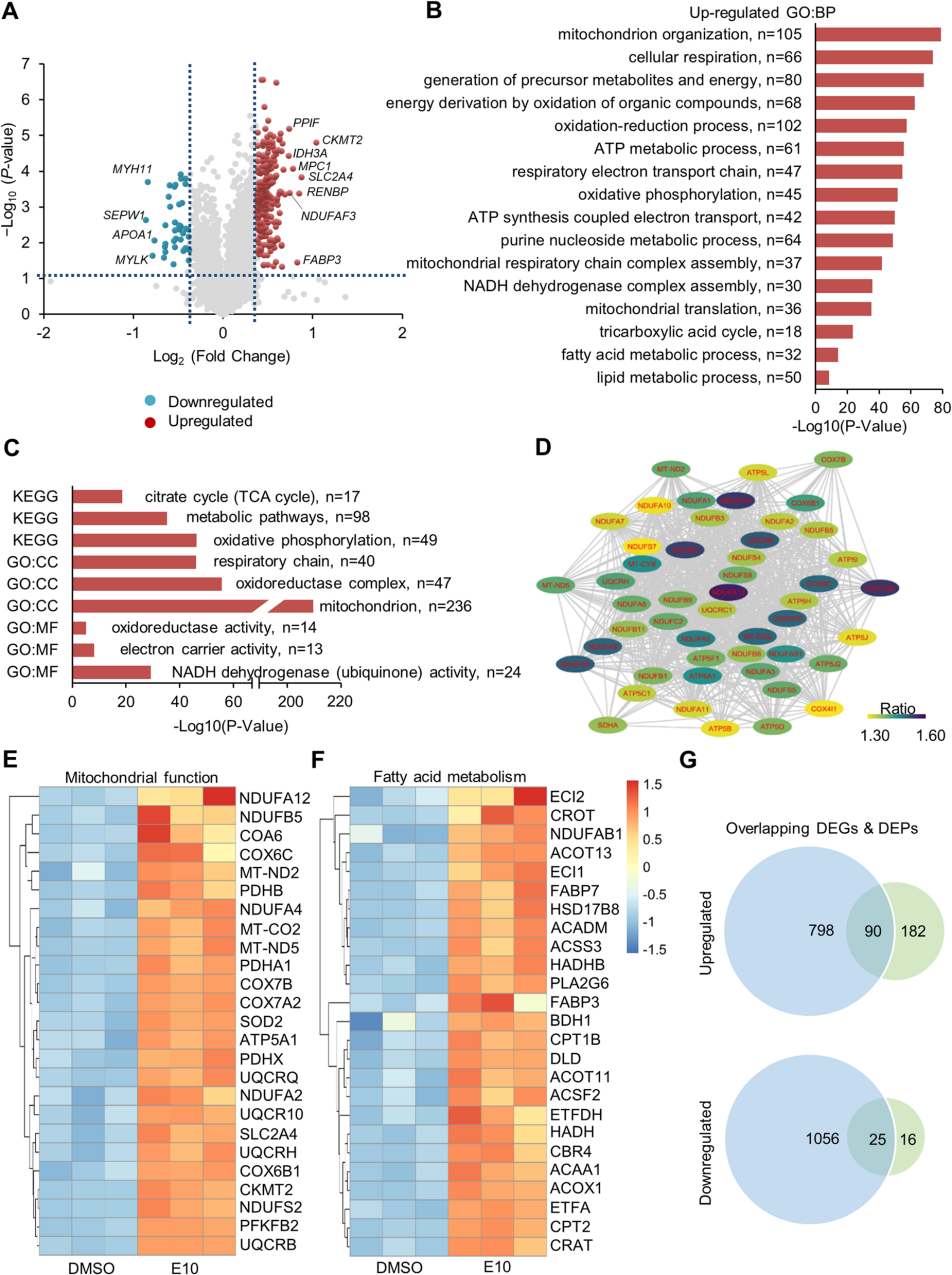
AMPK activation alters the expression of proteins in hiPSC-CMs identified by proteomics analysis. **A** Volcano plot illustrating proteins with statistically significant abundance differences between E10-treated and DMSO-treated hiPSC-CMs (*n* = 3 cultures). **B** Upregulated GO terms of biological processes. n refers to the number of proteins found in each GO term. **C** Upregulated pathways based on KEGG analysis and upregulated GO terms of cellular compartment (CC) and molecular function (MF). n refers to the number of proteins found in each pathway/GO term. **D** Protein interaction network of the upregulated proteins that participate in the oxidative phosphorylation pathway. Heatmaps showing the key upregulated proteins in E10-treated hiPSC-CMs versus DMSO-treated hiPSC-CMs associated with **E** mitochondrial function and **F** fatty acid metabolism. **G** Venn diagram showing the number of overlapping upregulated and downregulated genes (DEGs) and proteins (DEPs) in E10-treated hiPSC-CMs versus DMSO-treated hiPSC-CMs. DMSO, Dimethyl sulfoxide and E10, EX229 at 10 µM

To further examine the effect of AMPK activator on hiPSC-CM maturation, we performed quantitative proteomic analysis on 3D hiPSC-CMs treated with E10 vs. DMSO for 7 days. Of 5113 proteins analyzed, E10 treatment resulted in 272 upregulated and 41 downregulated proteins (absolute fold change > 1.3, *P* < 0.05) (Fig. 6A).

GO term analysis was performed using the differentially expressed proteins. Notably, in E10-treated hiPSC-CMs, top upregulated GO terms of biological process (BP) were associated with mitochondrial function and fatty acid metabolism (Fig. 6B). Based on cellular component (CC) and molecular function (MF), top GO terms were also related to mitochondrial metabolism (Fig. 6C). KEGG pathway analysis of the upregulated proteins in the E10-treated cells indicated upregulation of pathways associated with oxidative phosphorylation, metabolic pathways and citrate cycle (Fig. 6C). In addition, fatty acid degradation, cardiac contraction and PPAR signaling were also present in the upregulated KEGG pathways.

The String protein–protein interaction analysis identified a large number of differentially expressed proteins that are involved in the oxidative phosphorylation pathway. The high connectivity among these proteins further indicated their important roles in oxidative phosphorylation (Fig. 6D).

Detailed examination of the upregulated differentially expressed proteins identified the key proteins associated with mitochondrial biogenesis, TCA cycle and oxidative phosphorylation (Fig. 6E). These upregulated proteins included eight enzymes that catalyze the TCA cycle—CS, ACO2, IDH3A, IDH3B (isocitrate dehydrogenase (NAD(+)) 3 non-catalytic subunit beta), IDH3G (isocitrate dehydrogenase (NAD(+)) 3 non-catalytic subunit gamma), OGDHL (oxoglutarate dehydrogenase L), OGDH, DLST, DLD, SUCLG1 (succinate-CoA ligase GDP/ADP-forming subunit alpha), SUCLA2 (succinate-CoA ligase ADP-forming subunit beta), SDHA, SDHB, FH and MDH2 (malate dehydrogenase 2). In agreement with RNA-seq, E10 treatment upregulated SLC2A4, MT-CO2, CKMT2, PDHA1, PDHX, DLAT, DLD and another PDH E1 subunit PDHB.

In agreement with the observation in RNA-seq data, many important proteins in mitochondrial electron transport chains were upregulated in E10-treated cells. Specifically, we observed upregulated proteins in complex I—NADH dehydrogenase MT-ND2 and MT-ND5—and several NADH:ubiquinone oxidoreductase subunits—NDUFAF3, NDUFA12, NDUFA4, NDUFS2, NDUFA9, NDUFA5, NDUFAB1, NDUFA1, NDUFA3, NDUFS5, NDUFA8, NDUFC2, NDUFAF4, NDUFS8, NDUFB9, NDUFB1, NDUFB8, NDUFB11, NDUFS4, NDUFB3, NDUFB5, NDUFA2, NDUFA7, NDUFA11, NDUFA10 and NDUFS7. Upregulation of SDHA and SDHB in complex II was detected with upregulated *SDHB* being detected by RNA-seq as well. Additionally, UQCR subunits—UQCR10, UQCRB, UQCRC1 and UQCRH—in complex III and terminal components of the respiratory chain—COA6, COX6B1, COX6C, COX7A2 and COX7B—were upregulated. In line with our RNA-seq data, several subunits of ATP synthase F1 and F0 complex were upregulated, including ATP5A1, ATP5B, ATP5C1, ATP5O (ATP5PO, ATP synthase F1 subunit OSCP), ATP5H (ATP5PD, mitochondrial F0 complex, subunit D), ATP5I (ATP5ME, mitochondrial F0 complex, subunit E), ATP5L (ATP5MG, mitochondrial F0 complex, subunit G) and ATP5J (ATP5PF, mitochondrial F0 complex, subunit F). Also, E10-treated cells had increased expression of several solute carriers (SLC) family 25 members, including SLC25A24, SLC25A11, SLC25A5, SLC25A13 and SLC25A4. These results indicated an increased capacity of electron translocation for respiration and ATP synthesis in E10-treated cells.

Upregulation of many important proteins involved in fatty acid metabolism was also indicated. These included fatty acid-binding proteins FABP3 and FABP7, which are the intracellular fatty acid-binding proteins that regulate the uptake and transport of long-chain fatty acids into cells. The expression of SLC27A4 also increased, suggesting enhanced capacity of fatty acid uptake in E10-treated cells upon entry in cells and within cells. E10-treated cells had increased expression of acyl-CoA synthases (ACSs, which catalyze the process of fatty acid activation into fatty acyl-CoAs), including long-chain family members ACSL1, ACSL3 and short-chain family members ACSS2, ACSS3. Intriguingly, we found the upregulation of CPT1B (carnitine palmitoyltransferase 1B), carnitine acyl-carnitine translocase SLC25A20 and CPT2 (carnitine palmitoyltransferase 2). All of which are essential proteins in transporting long-chain fatty acyl-CoAs from the cytoplasm into mitochondria. Notably, E10-treated cells had upregulated MYLCD (malonyl-CoA decarboxylase, which catalyzes the breakdown of malonyl-CoA that inhibits CPT1, thus increasing the rate of CPT1-mediated fatty acid transport). E10 treatment also resulted in the upregulation of several enzymes which are essential to FAO process, including ACOX1 (acyl-CoA oxidase 1), ACADM, HADHA (hydroxyacyl-CoA dehydrogenase trifunctional multienzyme complex subunit alpha), HADHB and HADH (hydroxyacyl-CoA dehydrogenase). In addition, CROT (carnitine O-octanoyltransferase) and CRAT (carnitine O-acetyltransferase), BDH1 (3-hydroxybutyrate dehydrogenase 1) and ACAA1 (acetyl-CoA acyltransferase 1) involved in peroxisomal fatty acid oxidation as well as the key enzymes EC1 and EC2 in β-oxidation of unsaturated fatty acids were also upregulated. Collectively, the upregulation of the above essential proteins in E10-treated cells demonstrated increased consumption of fatty acid for the cell energy demand. Furthermore, comparison of the RNA-seq with proteomic data revealed a subset of overlapping genes and proteins that were differentially expressed in E10-treated cells. Specifically, 90 of them were upregulated and 25 were downregulated, respectively (Fig. 6G).

Together, these results revealed that E10 upregulated key regulators of mitochondrial function and FAO, which was consistent with improved metabolic maturation in E10-treated 3D hiPSC-CMs.

### Metabolomics identified enhanced energy-related metabolic pathways in E10-treated hiPSC-CMs

**Fig. 7:**
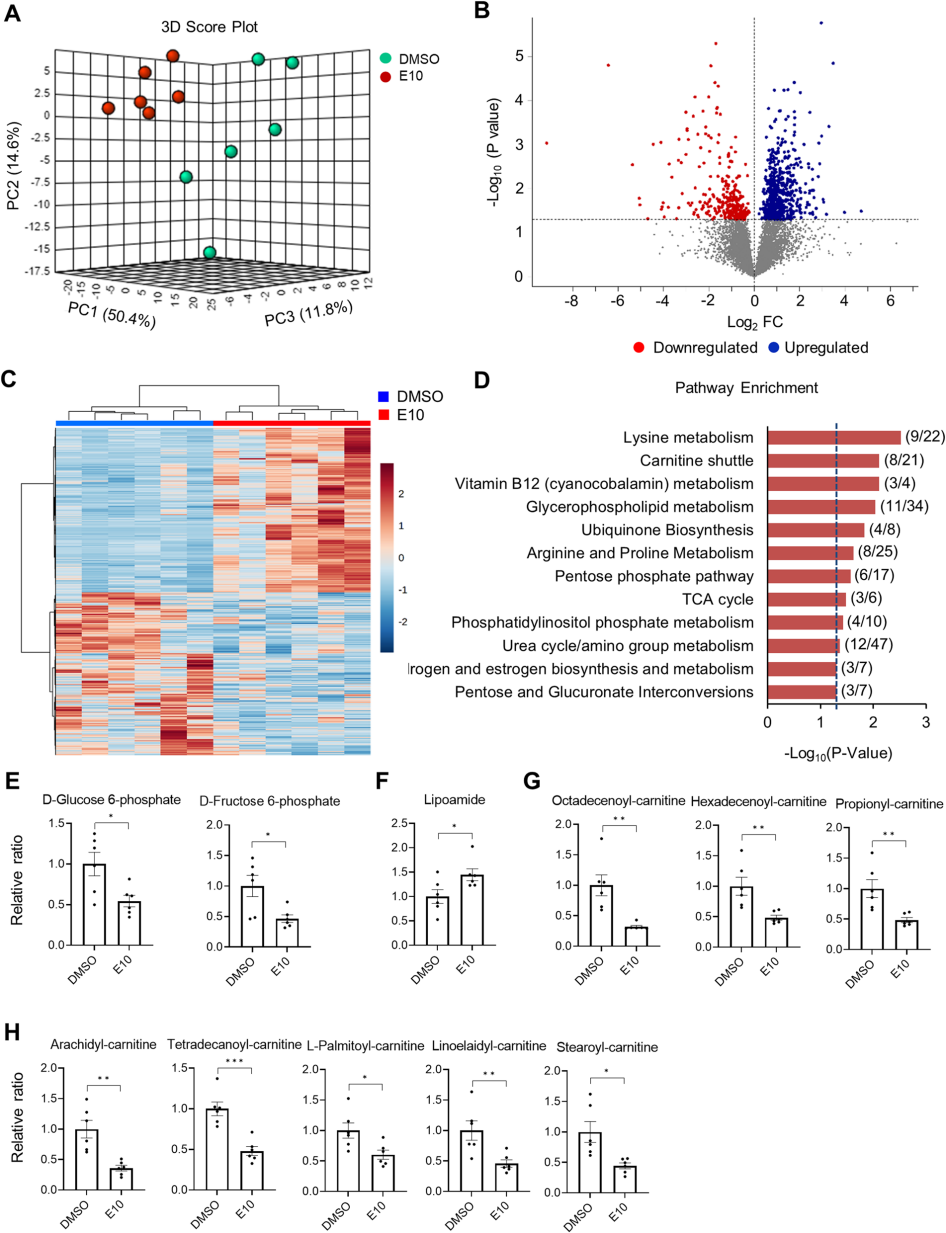
Metabolomics analysis identifies enhanced energy-related metabolic pathways in E10-treated hiPSC-CMs. **A** Three-dimensional principal component analysis (PCA) score plot based on metabolite abundance measured as ion peak intensities showing a significant separation between E10-treated and DMSO-treated hiPSC-CMs (*n* = 6 cultures). **B** Volcano plot illustrating discriminatory metabolites between E10-treated and DMSO-treated hiPSC-CMs. **C** Hierarchical clustering using the top 600 discriminatory features. **D** Enriched metabolic pathways using the 84 discriminatory metabolites. Significantly affected pathways were identified based on negative Log_10_ (P value < 0.05) and comprising of ≥ 3 metabolites per pathway. Comparison of **E** intermediates in glycolysis pathway, **F** lipoamide in TCA cycle, intermediates in carnitine shuttle including, **G** long-chain and **H** medium- or short-chain acyl-carnitines between E10-treated and DMSO-treated hiPSC-CMs. Data are presented as mean ± SEM. *P < 0.05; **P < 0.01; ***P < 0.001 and ****P < 0.0001 (two-sided unpaired t-test)

To gain additional understanding of the metabolic differences associated with AMPK activation, we performed untargeted metabolomic analysis using HILIC (ultra-high-resolution mass spectrometry with hydrophilic interaction liquid chromatography) to compare the metabolic profile of E10-treated hiPSC-CMs with their DMSO-treated counterparts. Of the 9903 features analyzed, 917 features significantly changed in E10-treated hiPSC-CMs from DMSO controls (*P* < 0.05). Principal component analysis (PCA) of the metabolic profiles between E10-treated hiPSC-CMs and DMSO controls showed a clear separation of metabolome between the two cultures (Fig. 7A). Unsupervised two-way hierarchical analysis of the significant features confirmed the distinction of metabolic profiles between the E10-treated hiPSC-CMs and DMSO controls (Fig. 7C, Metabolic feature table in Additional file 3).

To identify the significantly affected pathways, we performed pathway enrichment analysis on the 917 metabolic features that were different between the groups and identified 12 pathways that were enriched in E10-treated hiPSC-CMs. Top pathways included multiple mitochondria and energy metabolism-related activities, including lysine metabolism, carnitine shuttle, vitamin B12 (cyanocobalamin) metabolism, glycerophospholipid metabolism, ubiquinone biosynthesis, arginine and proline metabolism, pentose phosphate pathway, TCA cycle, phosphatidylinositol phosphate metabolism, urea cycle/amino group metabolism, androgen and estrogen biosynthesis and metabolism, pentose and glucuronate interconversions (Fig. 7D).

In E10-treated cells, two intermediates of the glycolysis pathway, D-glucose-6-phosphate and fructose-1,6-bisphosphate, were significantly decreased, while the level of D-glucose and pyruvate was not significantly different compared with DMSO-treated cells (Fig. 7E). In addition, the level of lipoamide was significantly increased in E10-treated cells (Fig. 7F). Lipoamide acts as an essential cofactor for mitochondrial 2-ketoacid dehydrogenases that are key enzymes in TCA cycle, which include PDH, OGDH and branched-chain ketoacid dehydrogenase (BCKDH) [20–22]. Combined results in  RNA-seq and Proteomics on upregulated PDH, OGDH and BCKDH, the increased level of lipoamide further suggested an enhanced TCA cycle in E10-treated hiPSC-CMs based on our findings in GO and KEGG pathway analysis.

Among the upregulated pathways, carnitine shuttle system is responsible for the transport of fatty acids into mitochondria for subsequent β-oxidation. Upon cellular entry, fatty acids are activated into fatty acyl-CoAs by ACSs followed by conjugation to carnitine forming fatty acyl-carnitines catalyzed by CPT1B and located in the outer mitochondrial membrane. Fatty acyl-carnitines then diffuse across the mitochondrial membrane by action of carnitine acyl-carnitine translocase (SLC25A20) located on inner mitochondrial membrane. Once within mitochondria, carnitine is released from fatty acyl-carnitines by CPT2 and transferred back to cytoplasm, while fatty acyl-CoAs enter β-oxidation. Interestingly, E10 treatment significantly reduced the level of fatty acyl-carnitines in E10-treated cells compared to DMSO control (Fig. 7G and H). Specifically, we found marked, lower levels of short-chain fatty acyl-carnitine propionyl-carnitine, two medium-chain fatty acyl-carnitines (octadecenoyl-carnitine and hexadecenoyl-carnitine) and five long-chain fatty acyl-carnitines (L-palmitoyl-carnitine, linoelaidyl-carnitine, stearoyl-carnitine, arachidyl-carnitine and myristoyl-carnitine). Together, they imply elevated fatty acid β-oxidation in E10-treated cells.

Overall, these findings suggested AMPK activation-induced metabolic changes and promoted metabolic flux in energy-related metabolisms in hiPSC-CMs. These results demonstrate consistency with our findings in RNA-seq and proteomics analyses and reveal the upregulated expression profile of key regulators in carnitine shuttle system which remarkably facilitated fatty acids transportation and β-oxidation.

### AMPK activator-treated hiPSC-CMs enabled pathological modeling

**Fig. 8:**
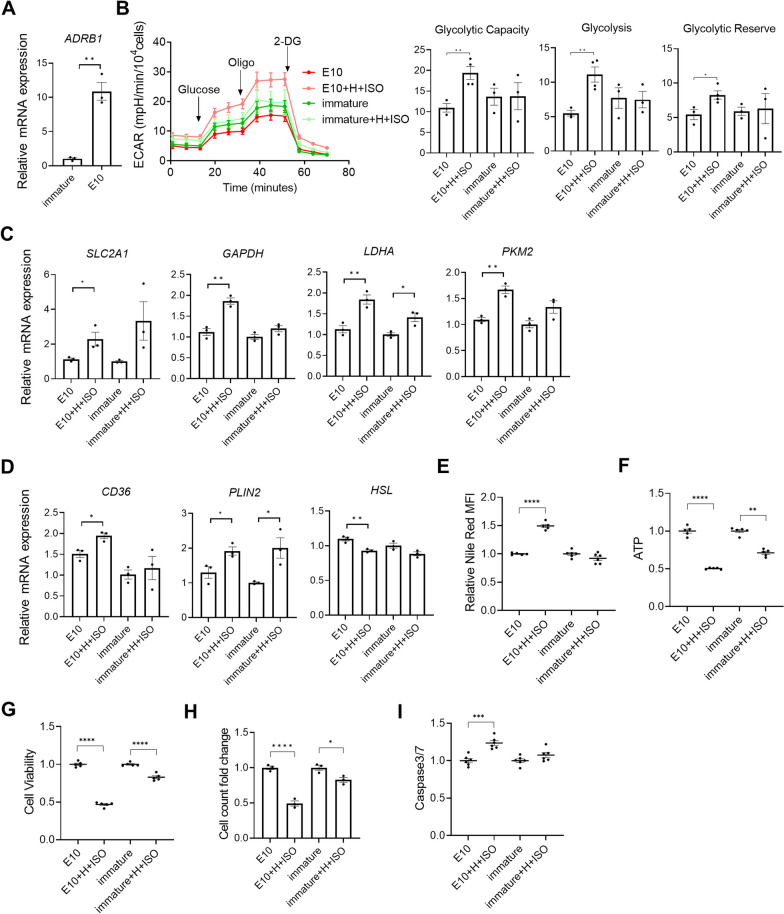
E10-treated hiPSC-CMs enhance pathological modeling. E10-treated and immature (without E10 treatment) hiPSC-CMs were subjected to pathological stimulation (H + ISO) and analyzed for pathological outcomes. **A** Expression of *ADRB1* (*n* = 3 cultures). **B** Representative kinetics of ECAR and quantification of glycolysis, glycolytic capacity and glycolytic reserve (*n* = 4 cultures). **C** Expression of genes associated with glycolysis and **D** biosynthesis of triacylglycerol *CD36*, *PLIN2* and *HSL* (*n* = 3 cultures). **E** Quantitative analysis of Nile red staining by ArrayScan (*n* = 5 cultures). **F** Relative ATP content (*n* = 7 cultures), **G** relative cell viability (*n* = 5 cultures), **H** cell count (*n* = 3 cultures) and **I** caspase 3/7 activity (*n* = 6 cultures). Data are presented as mean ± SEM. *P < 0.05; **P < 0.01; ***P < 0.001 and ****P < 0.0001 (two-sided unpaired t-test). 2-DG, 2-Deoxy-d-glucose; CD36, cluster of differentiation 36; E10, EX229 at 10 µM; ECAR, Extracellular acidification rate; GAPDH, Glyceraldehyde-3-phosphate dehydrogenase; H, Hypoxia; ISO, Isoproterenol; HSL, Lipase E, hormone-sensitive type; LDHA, Lactate dehydrogenase A; Oligo, Oligomycin; PKM2, Pyruvate kinase M1/2; PLIN2, Perilipin 2 and SLC2A1, Solute carrier family 2 member 1

Based on aforementioned assessments of multiple features associated with metabolic maturation and extensive omic analyses, we concluded that E10 treatment could drive significantly increased FAO and metabolic maturation in 3D hiPSC-CMs. In addition, the expression of *ADRB1* (adrenergic receptor B1) was significantly upregulated in AMPK-activated hiPSC-CMs (Fig. 8A). Given that the metabolic switch from FAO to glycolysis is associated with heart failure [23], we examined if E10-treated 3D hiPSC-CMs could model pathological response and recapitulate some of the changes related to heart failure including glycolysis enhancement, lipid accumulation and apoptosis. Three-dimensional hiPSC-CMs were first treated with E10 for 7 days to promote cardiomyocyte maturation. The cells were then treated with isoproterenol (100 µM), a small molecule adrenergic agonist to simulate pathological conditions for 6 days in hypoxic environment and low-glucose medium. The treatment with pathological stimuli increased glycolysis, glycolytic capacity and glycolytic reserve compared with no pathological stimuli in E10-treated cells, as detected by the extracellular acidification rate (ECAR; index of glycolytic activity) using the Seahorse assay (Fig. 8B). In contrast, the treatment of pathological stimuli did not alter glycolysis, glycolytic capacity and glycolytic reserve in immature cells (without E10 treatment) (Fig. 8B). Consistently, E10-treated cells but not immature cells had significant upregulation of the glycolysis-related genes including *SLC2A1*, *GAPDH*, *LDHA* and *PKM2* under the treatment with pathological stimuli compared with cells without the treatment with pathological stimuli (Fig. 8C). These results indicate that E10-treated hiPSC-CMs had increased glycolysis when challenged with pathological stimuli, a pathological response associated with heart failure [7].

The pathological stimuli also induced the upregulation of triacylglycerol synthesis-associated gene *CD36* (cluster of differentiation 36) and *PLIN2* (Perilipin 2; a marker of lipid accumulation) in E10-treated 3D hiPSC-CMs but not in immature cells (Fig. 8D). In addition, the expression of *HSL* (*LIPE*; Lipase E, hormone-sensitive type) that hydrolyzes stored triglycerides to free fatty acids was downregulated in E10-treated hiPSC-CMs when challenged with pathological stimuli (Fig. 8D). Consistently, an increased accumulation of lipid droplets was observed in E10-treated hiPSC-CMs when challenged with pathological stimuli as detected by ArrayScan analysis of Nile red staining (Fig. 8E). Increased accumulation of lipid droplets is also a pathological response associated with heart failure [24].

In addition to the change in glycolysis and lipid accumulation, the pathological stimuli resulted in significantly increased cell death (Fig. 8F–I). Following the treatment of pathological stimuli, E10-treated cells had twofold decreased ATP content while immature hiPSC-CMs had 29% decreased ATP content compared with parallel cultures without the treatment of pathological stimuli. Similarly, the treatment of pathological stimuli resulted in higher magnitude of decrease in cell viability and cell counts in E10-treated cells compared with immature cells. In addition, increased caspase 3/7 activity was observed in E10-treated cells when challenged with the pathological stimuli.

These results suggest that E10-treated hiPSC-CMs responded to pathological stimuli, which was not achievable in immature hiPSC-CMs. Thus, E10-treated hiPSC-CMs have advantages for pathological modeling.

## Discussion

In this study, we demonstrated pharmacological agent-induced AMPK activation could drive hiPSC-CMs to a more mature stage with improved metabolic, structural and functional properties. The maturation treatment was conducted in size-controlled cardiac spheres which are scalable and suitable for high-throughput assays. Three-dimensional hiPSC-CMs treated with AMPK activators had increased ATP content, mitochondria content, mitochondrial membrane potential and mitochondrial function, as well as increased fatty acid uptake and FAO, suggesting that these cells were metabolically more mature. In contrast, inhibition of AMPK by pharmacological treatment and genetic modification decreased mitochondrial function and abolished the AMPK activator improved mitochondrial function in hiPSC-CMs. In addition, we observed an improved structural development after AMPK activator treatment including the increased sarcomere length, cell area, perimeter and length to width ratio. AMPK activator-treated hiPSC-CMs also had enhanced calcium transient kinetics and higher velocities of maximum contraction and relaxation, which are functional features of more mature cardiomyocytes. RNA-seq, proteomic and metabolomic analyses revealed a distinct molecular signature associated with more mature cardiomyocytes in AMPK activator-treated 3D hiPSC-CMs. Furthermore, the resulting more mature hiPSC-CMs enable pathological modeling that was not achievable in immature hiPSC-CMs. Together, this study provides valuable insights into the strategies that help to achieve hiPSC-CM maturation through metabolic regulation.

During our study, other groups reported that the treatment of differentiated cells in 2D cultures with AMPK activator AICAR increased metabolic maturation of hiPSC-CMs [25, 26]. Compared with the previous publications, our study highlights the following new findings. (1) We found that AMPK activation enabled pathological modeling. (2) We identified an AMPK activator that is more potent than the one used in the previous publications. (3) We found that AMPK activation not only promoted metabolic maturation but also functional maturation. (4) We characterized the molecular action of AMPK activation through comprehensive transcriptomics, proteomics and metabolomics analyses. (5) We used human heart tissue as a control for molecular characterization. (6) We confirmed the role of AMPK by inhibition of AMPK through both pharmacological and genetic methods. (7) Three-dimensional cultures of cardiac spheres generated highly enriched cardiomyocytes which allow more rigorous investigation of the effect of AMPK activation on metabolic maturation.

Our cardiac differentiation approach and 3D culture produced hiPSC-CMs at high purity, serving as excellent starting materials for examining the effect of AMPK activators on cardiomyocyte maturation. We believe that this is a critical and rigorous step before evaluating culture conditions for promoting cardiomyocyte maturation, since if the assessment of mitochondrial function is done in bulk cultures, it is possible that changes in mitochondrial function simply reflect changes in cardiomyocyte purity in cultures (cardiomyocytes have higher mitochondrial content than non-cardiac cells). The cardiac spheres used in our study were generated by microscale tissue engineering [27]. The advantage of this technology is that it allows for the generation of 3D multicellular aggregates with controlled sphere sizes. An optimal sphere size is important to overcome the limitation on molecule diffusion into 3D cultures to allow cells to receive molecules in the medium effectively. Typically, cardiac spheres generated by microscale tissue engineering do not contain necrotic centers suggesting sufficient diffusion of nutrient molecules to the center of spheres [12].

Our approach for screening AMPK activators for promoting metabolic maturation is effective by using assays of mitochondrial features including ATP content, mitochondrial membrane potential and mtDNA:nDNA ratio. These three high-throughput assays complemented each other and ensured the selection of optimal drugs and doses for our study. Among 4 AMPK activators, we selected A-769662 and EX229 with each activator at two doses for further evaluation in 3D hiPSC-CMs and found consistent outcomes compared with our initial screening. We also found the outcomes of ATP content, mitochondrial membrane potential and mtDNA:nDNA ratio correlated well with mitochondrial functional measurement by Seahorse Mito Stress test.

Both A-769662 and EX229 have been reported as a potent and specific pharmacological activator of AMPK. A-769662 can allosterically activate AMPK containing the β1 isoform and inhibit dephosphorylation of Thr172 on α subunits [28, 29]. A-769662 binds to the cleft between the catalytic α subunits and the regulatory β subunits and its function highly depends on the autophosphorylation site Ser108 within the β subunit carbohydrate-binding module (CBM). Mutation of the Ser108 on β1 subunit can almost completely abolish the activation of AMPK [30–33]. EX229 is a cyclic benzimidazole derivative that binds to the same site of AMPK as A-769662 but is five to tenfold more potent than A-769662 in cell-free assays [34]. Consistently, we also found that EX229 was more potent in promoting hiPSC-CM maturation than A-769662.

Treatment of 3D hiPSC-CMs with EX229 also increased fatty acid uptake and FAO rates, which are key features of metabolic maturation of cardiomyocytes since immature cardiomyocytes rely on glycolysis as the primary energy source. As cardiomyocytes mature, mitochondrial content significantly increases to meet the high energetic demands, and consequently, FAO becomes as the dominant energy provider due to the increased mitochondrial function and oxidative capacity. The increased FAO in hiPSC-CMs is consistent with our findings on mitochondrial maturation induced by the treatment with EX229 based on comprehensive and complementary measurements. High-content imaging by ArrayScan for TMRM and TOM20 together with qPCR analysis of the mtDNA/nDNA in AMPK activator-treated hiPSC-CMs demonstrated that AMPK activation significantly increased mitochondrial membrane potential and content of hiPSC-CMs, which was further confirmed by flow cytometry analysis of MitoTracker Red and TOM20. Furthermore, the elevated mitochondrial function was confirmed using the Seahorse Mito Stress assay, which displayed higher level of basal and maximal oxygen consumption, and ATP production. Such increased mitochondrial function is likely to satisfy the increased energy demands indicating a higher respiratory capacity and oxidative potential occurred in AMPK activator-treated hiPSC-CMs; consistently, higher level of ATP content was detected in these cells. In addition, transcriptomic and proteomic profiling also identified a large number of differentially expressed genes and proteins linked to metabolic maturity of cardiomyocytes including upregulation of proteins associated with mitochondrial biogenesis and oxidative phosphorylation, indicating the robustness of metabolic modulation via AMPK activation. Since mitochondrial dynamics can regulate mitochondrial function, it will be of interest to investigate the effect of AMPK activators on mitochondrial morphology using transmission electron microscope.

The role of AMPK activation in mitochondrial maturation of hiPSC-CMs was also confirmed through the treatment with a pharmacological inhibitor of AMPK, Compound C, or with siRNA that targets AMPK. Both Compound C or siRNA knockdown of *AMPK* decreased key mitochondrial features including ATP content, mitochondrial membrane potential and mtDNA:nDNA ratio, as well as mitochondrial function detected by the Seahorse Mito Stress test. In addition, both Compound C and siRNA knockdown *AMPK* abolished the positive effect of AMPK activators.

We also found that metabolic maturation induced by AMPK activators was correlated with higher levels of structural and functional maturation. A hallmark of cardiomyocyte functional maturation is the development of Ca^2+^ handling and contractility. Immature cardiomyocytes differ in many ways in Ca^2+^ handling compared with mature cardiomyocytes, such as lower calcium transient amplitudes and slower rise/decay kinetics [3]. AMPK activator-treated hiPSC-CMs exhibited significantly improved calcium handling properties including significantly faster rate of rise and decay of Ca^2+^ transients. RNA-seq also revealed that AMPK activator-treated hiPSC-CMs had increased expression of several genes that are important in regulating Ca^2+^ handling and electrophysiology, such as *PLN*, *KCNJ2*, *ATP2A2*, *SCN1B* and *CACNG4*. *KCNJ2* is the major inward-rectifier type potassium channel that establishes and maintains resting membrane potential and cell excitability. *PLN* plays a crucial role in regulating Ca^2+^ cycling in cardiomyocytes [35]. Many DEGs and GO terms identified in AMPK activator-treated hiPSC-CMs were also identified in pediatric heart tissues when these samples were compared with DMSO-treated hiPSC-CMs. However, we also note that a large number of genes were differentially expressed when AMPK activator-treated hiPSC-CMs were compared with pediatric heart tissues, suggesting that the maturation level of AMPK activator-treated hiPSC-CMs differed from that of mature cardiomyocytes in pediatric heart. To reach higher levels of maturation, further study is needed to examine the effect of combining AMPK activation with other methods such as electromechanical stimulation [36] and co-culture with non-cardiac cells [37] on hiPSC-CM maturation. In addition, DEGs identified in E10-treated cells are likely related to the effects of E10 treatment on cellular processes such as metabolism, signaling pathways or cell differentiation. Further analysis and experimentation would be helpful to understand molecular mechanisms underlying E10-promoted cardiomyocyte maturation.

The resulting more mature hiPSC-CMs could serve as a valuable model for understanding the underlying mechanisms of cardiac pathological conditions in humans. hiPSC-CMs have drawn extensive attention in in vitro disease modeling due to their remarkable advantages over animal models in overcoming physiological difference among species, recapitulating complexities of human cardiomyocytes. More mature hiPSC-CMs with improved metabolic maturation are particularly helpful for the study of cardiac metabolic remodeling during disease. Under normal conditions, FAO by mitochondria provides a main energy source in mature cardiomyocytes. Under pathological conditions, cardiomyocytes undergo metabolic remodeling by increasing glycolysis. Such disease phenotype is difficult to model using immature hiPSC-CMs since these cells have limited capacity of FAO, and instead, they use glycolysis as a main energy source. Indeed, we found that AMPK activator-treated hiPSC-CMs were able to respond to pathological stimuli and to produce disease phenotypes including glycolysis enhancement, lipid accumulation and cell death, which were not achievable in untreated immature hiPSC-CMs. In our pathological modeling experiments, we used isoproterenol to induce disease phenotypes in hiPSC-CMs. Although isoproterenol can induce many aspects of the pathophysiology of heart failure and is commonly used to study cardiac defects in animal models, this model has limitations to fully recapitulate clinical outcomes. In addition, future study can help establish if the maturation treatment also improves the use of hiPSC-CMs in modeling other cardiac diseases such as hypertrophic cardiomyopathy and dilated cardiomyopathy [38]. Nevertheless, our results suggest that higher levels of metabolic maturity rendered hiPSC-CMs a unique capacity of modeling the key events in cardiomyocytes during heart failure, highlighting the importance of cardiomyocyte maturation.

## Conclusions

In summary, our study demonstrated that hiPSC-CMs could be driven to a more mature stage by activating AMPK using a pharmacological agent-induced strategy. The AMPK activator-treated hiPSC-CMs allowed us to conduct pathological modeling study that was not achievable in non-modulated hiPSC-CMs. In addition, more mature hiPSC-CMs will also advance broad applications of these cells in regenerative medicine, modeling of other cardiac diseases and cardiotoxicity. Our approaches used in screening AMPK activators paved an effective way for the future drug and dose identification in pharmacological drug-induced hiPSC-CM maturation. Our findings will also facilitate future efforts on large-scale production of mature hiPSC-CMs, given that our cardiac sphere culture system has advantage for scale-up suspension culture [39].

### Supplementary Information

**Additional file 1.** Supplemental results, methods, tables and figures.

**Additional file 2.** Beating cardiac spheres.

**Additional file 3.** Metabolic feature table.

## Data Availability

The RNA-seq data were deposited at the GEO database under accession code GSE203480. The proteomic data were deposited in the public MassIVE database with the identifier MSV000087351.

## Abbreviations

3D: Three-dimensional
A100: A-769662 at 100 µM
A200: A-769662 at 200 µM
E10: EX229 at 10 µM
E50: EX229 at 50 µM
ECAR: Extracellular acidification rate
FAO: Fatty acid oxidation
GO: Gene Ontology
HILIC: Ultra-high-resolution mass spectrometry with hydrophilic interaction liquid chromatography
hiPSC: Human-induced pluripotent stem cell
hiPSC-CMs: Cardiomyocytes derived from human-induced pluripotent stem cells
LC–MS–MS: Liquid chromatography with tandem mass spectrometry
LV: Heart tissue samples from pediatric left ventricle
OCR: Oxygen consumption rate
RNA-seq: RNA-sequencing
siRNA: Small interfering RNA
