# Supplementary material for: AMPK activator-treated human cardiac spheres enhance maturation and enable pathological modeling
Source: Stem Cell Res Ther. 2023 Nov 8;14:322. doi: 10.1186/s13287-023-03554-7 (PMC10633979; doi:10.1186/s13287-023-03554-7)
Supplement: Supplementary file 1 — Additional file 1. Supplemental results, methods, tables and figures. [file 13287_2023_3554_MOESM1_ESM.pdf]

SUPPLEMENTARY MATERIAL

SUPPLEMENTARY RESULTS

AMPK activators dose screening

We generated hiPSC-CMs from IMR90 and SCVI273 hiPSC lines using a well-established cardiac differentiation protocol [1]. We treated hiPSC-CMs from both cell lines with four AMPK activators including EX229, A-769662, AICAR and metformin for two weeks and determined the optimal dose of each activator by checking their effects on ATP content, mitochondrial membrane potential and mtDNA:nDNA ratio. These activators at high concentrations, EX229 (100 μM), A-769662 (300 μM), AICAR (1 mM, 5 mM) and metformin (10 mM, 20 mM), induced cell death and were eliminated in the screening process. In IMR90 hiPSC-CMs, the treatment with EX229 at 1 μM, 10 μM and 50 μM significantly increased the ATP content, mitochondrial membrane potential and mtDNA:nDNA ratio in a dose-dependent manner and reached to the maximum at 50 μM in all three measured assays (Figure S1A-C). There was significant difference between 50 μM with DMSO, 0.1 μM, 1 μM but no significant difference with 10 μM treatment. The incubation with A-769669 showed a similar dose-dependent increase in ATP content, mitochondrial membrane potential and mtDNA:nDNA ratio and achieved the significantly higher levels at high doses 100 μM and 200 μM, which had no significant difference in between (Figure S1A, S1B and S1D). The incubation with AICAR at all doses significantly increased the level of ATP content, and there was no significant difference among these doses (Figure S1A). AICAR at 500 μM significantly increased mitochondrial membrane potential (Figure S1B). However, the incubation with AICAR did not increase mtDNA:nDNA ratio at any dose (Figure S1E). Similarly, the incubation with Metformin significantly increased the production of ATP but did not induce significant increases in mitochondrial membrane potential and mtDNA:nDNA ratio and the effect of Metformin on ATP content among doses was not significant (Figure S1A, S1B and S1F).

In SCVI273-CMs, the incubation with EX229 significantly increased the level of ATP content at all doses. The increase in mitochondrial membrane potential could be detected with all doses but only the effect of 50 μM was significantly different compared with DMSO. The incubation with EX229 also increased mtDNA:nDNA ratio, but only its effect in ND1/SDHA at 0.1 μM, 1 μM, 10 μM and ND1/LPL at 10 μM was evaluated as significant (Figure S2A through S2C). The incubation with A-769662 increased the level of ATP and mitochondrial membrane potential at all doses, but its effect on mitochondrial membrane potential was not significantly different compared with DMSO. In addition, A-769662 at 200 μM could significantly increase the mtDNA:nDNA ratio (Figure S2A, S2B and S2D). A significant increase in ATP content could be detected in the incubation with AICAR at all doses. AICAR could also increase mitochondrial membrane potential and its effect at 10 μM and 100 μM was significant. However, the incubation with AICAR failed to achieve an increase on mtDNA:nDNA ratio at all doses (Figure S2A, S2B and S2E). The incubation with Metformin significantly increased ATP content at all doses, whereas only 100 μM increased the mitochondrial membrane potential significantly. The incubation with Metformin tended to increase the mtDNA:nDNA ratio, but the increase was not significant (Figure S2A, S2B and S2F).

Overall, all four activators significantly increased the ATP content after 14 days treatment. Only EX229 and A-769662 induced a significant increase in mitochondrial membrane potential and mtDNA:nDNA ratio compared to DMSO. The incubation with EX229 at 10 μM and 50 μM, and A-769662 at 100 μM and 200 μM, respectively, demonstrated a superior effect on all three measured assays.

Treatment of 3D hiPSC-CMs did not alter cardiomyocyte purity

In 3D differentiation cultures, hiPSC-CM purity was consistently high as determined by flow cytometry for cTnT/α-actinin double staining and high-content imaging analysis for NKX2-5/α-

actinin staining by ArrayScan. On differentiation day 14, cultures derived from IMR90 hiPSCs contained 94% to 96% $cTnT^+/\alpha$ -actinin$^+$ cells as detected by flow cytometry analysis (Figure 1B) and 96% NKX2-5$^+$ cells and 96% a-actinin$^+$ cells as detected by ArrayScan (Figure 1C). Following the treatment of AMPK activators for 7 days (Figure 1D) and 14 days (Figure S3A), proportions of cells positive for NKX2-5 and α-actinin remained at high levels, similar to those in the cultures before the treatment. Therefore, treatment of 3D hiPSC-CMs with AMPK activators did not alter cardiomyocyte purity.

SUPPLEMENTARY METHODS

Human induced pluripotent stem cell culture, cardiac differentiation, and generation of cardiac spheres

IMR90 hiPSCs (WiCell) and SCVI273 hiPSCs (Stanford Cardiovascular Institute) were maintained at 37°C and 5% CO_2 on Matrigel (BD Biosciences, San Jose, CA)-coated 6-well plate and fed daily with mTeSR1 medium (Stem Cell Technologies, Vancouver, Canada). For cardiac differentiation, hiPSCs at 70-80% confluence were dissociated using Versene/EDTA (Thermo Fisher Scientific, Waltham, MA) and plated onto Matrigel-coated 12-well plates at a density of 4 X 10^5 cells/well and cultured for two days in mTeSR1 medium prior to the medium change with RPMI medium containing 2% B27 insulin-free (Thermo Fisher Scientific, Waltham, MA) supplemented with 100 ng/mL recombinant human activin A (R&D Systems, Minneapolis, MN) (Day 0). The cells were treated with activin A for 24h followed by 3 days treatment with 10 ng/mL recombinant human bone morphogenic protein-4 (BMP4; R&D Systems, Minneapolis, MN) in RPMI medium with 2% B27 insulin-free (Day 1 to 4). On Day 4, medium was replaced with RPMI medium containing B27 with insulin.

To generate cardiac spheres, we used a microtissue engineering method as described [2]. Specifically, cells were dissociated on Day 5 using 0.25% trypsin/EDTA (Thermo Fisher Scientific, Waltham, MA) and transferred into single wells of AggreWell 400 plates (Stem Cell Technologies, Vancouver, Canada) at 1.5 X 10^4 cells/microwell in RPMI medium containing B27 with insulin supplemented with 10 µM Rock inhibitor Y-276322 (Stemgent, Cambridge, MA) to prevent cell death. After 24 h, cardiac spheres were harvested and transferred into low attachment 35 mm dishes in RPMI+B27 medium with insulin. The cardiac spheres were maintained in suspension culture with medium change every other day until Day 14.

AMPK activators dose screening

To screen doses of AMPK activators, at differentiation Day 14, hiPSC-CMs were cultured in cardiomyocyte maturation medium [DMEM, 10% fetal bovine serum, 2 mM L-glutamine and 1% penicillin-streptomycin, 0.1 mM non-essential amino acids, 100 µM oleic acid (Sigma-Aldrich, St. Louis, MO) and 50 µM palmitic acid (Cayman Chemical, Ann Arbor, MI) supplemented with the following AMPK activators: (1) A-769662 at 1 µM; 10 µM; 100 µM; 200 µM; 300µM; (2) AICAR at 10 µM, 100 µM, 500 µM, 1 mM, and 5 mM; (3) metformin at 10 µM, 100 µM, 1 mM, 10 mM, and 20 mM; and (4) EX229 at 0.1 µM, 1 µM, 10 µM, 50µM, and 100µM. A-769662, AICAR and metformin were obtained from Cayman Chemical and EX229 was obtained from Selleckchem. All drugs were reconstituted according to manufacturer's instructions in DMSO. DMSO was used a solvent control for the maturation treatment. AMPK activators supplemented maturation medium was refreshed every other day for 14 days. The optimal dose of each activator was determined by checking mitochondrial membrane potential, mitochondrial DNA (mtDNA) to nuclear DNA (nDNA) ratio and ATP content, as described below.

Immunocytochemical analysis

Cardiac spheres at day 28 were dissociated with 0.25% trypsin/EDTA and cultured on Matrigel-coated 96-well plates for one day for ArrayScan analysis. For confocal microscopy analysis, the

dissociated CMs were plated on Lab-Tek chamber slides (Thermo/Nunc) for one day culture. CMs were then fixed in 4% paraformaldehyde (PFA) for 15 min at room temperature. They were permeabilized with 0.1% Triton-X 100 (Sigma-Aldrich, St. Louis, MO) in PBS for 30 min at room temperature. Permeabilization was not performed when cells were immunostained for cell surface makers. After washing two times with PBS, cells were incubated in blocking buffer [5% normal goat serum (Sigma-Aldrich, St. Louis, MO) in 0.25% BSA/PBS] for 1 h at room temperature. Primary antibodies were diluted in 0.25% BSA/PBS and cells were cultured in diluted primary antibodies overnight at 4°C with gentle rotation. Primary antibodies used were listed in Supplementary Table 1.

High-content imaging analysis by Arrayscan

Images of stained cells were acquired and quantitatively analyzed using ArrayScan™ XTI Live High Content Platform. Twenty fields/well were imaged using a 10x objective. The Acquisition software Cellomics Scan (Thermo Fisher Scientific, Waltham, MA) was used to capture images and data analysis was performed using Cellomics View Software (Thermo Fisher Scientific, Waltham, MA). For the NKX2-5 assay, images were analyzed with a mask modifier for Hoechst and NKX2-5-positive cells restricted to the nucleus. The percentage of NKX2-5-positive cells in each treatment was used as readout. For detection of α-actinin, TMRM, TOM20 and Nile Red, images were analyzed with a mask modifier for Hoechst restricted to the nucleus. Spot threshold was set to 10 units and detection limit was set at 25 units. The percentage of α-actinin-positive cells and mean fluorescence intensity of TMRM, TOM20 and Nile Red were used as readout.

Quantitative RT-PCR (qRT-PCR) analysis

Total RNA was extracted from hiPSC-CMs using Aurum total RNA mini kit according to manufacturer's instructions. cDNAs were prepared from 1 µg RNA sample using the Superscript VILO cDNA synthesis kit (Thermo Fisher Scientific, Waltham, MA), and reaction mixture was incubated using a C1000 touch thermal cycler (Bio-Rad, Hercules, CA) as follows: 25°C for 10 minutes, 42°C for 2 hours and 25°C for 5 minutes. Reaction mixture was further diluted to 300 µL and 2 µL cDNA was subjected to qRT-PCR, which was performed in triplicates using a SYBR Green reaction master mix (Bio-Rad, Hercules, CA). mRNA levels were normalized to *GAPDH* or *TBP* mRNA levels. Primer sequences were shown in Supplementary Table 2.

Flow cytometry

The mitochondrial membrane potential was analyzed using the fluorescent dye MitoTracker Red (M7512; Thermo Fisher Scientific, Waltham, MA) that emits 599 nm light when accumulated in mitochondria. Cardiac spheres were dissociated with 0.25% Trypsin/EDTA and washed with PBS and labeled with 30 nM MitoTracker Red for 30 mins in pre-warmed 0.25% BSA/PBS at 37℃ in a 5% CO_2 incubator. Cells were then fixed with 4% PFA for 15 mins and analyzed by BD FACS Canto II (BD Biosciences, San Jose, CA). Forward versus side scatter quadrants were defined and at least 10,000 live cells were acquired.

Flow cytometry analysis of the purity and mitochondria content of hiPSC-CMs was performed following a protocol as described previously [2]. Purity was determined upon targeting cTnT and α-actinin while the mitochondria content analyzed using the antibody against TOM20. Briefly, cardiac spheres were dissociated into single cells with 0.25% Trypsin/EDTA. Cells were stained with Fixable Live/Dead Near-IR and fixed in 2% PFA, subsequently cells were permeabilized by incubating with absolute methanol on ice for 30 min and then blocked using 20% normal goat serum. Further, cells were stained with either conjugated antibody or sequentially stained with primary and secondary antibodies. Stained cells were analyzed by BD FACS Canto II or FACS Aria II by adjusting voltage and color compensation using appropriate excitation and detection channels. Forward versus side scatter quadrants were defined and at least 10,000 live cells were acquired. Finally, dot plots were generated upon data analysis using

FlowJo software to display the percentage of cell populations. All antibodies are listed in Supplementary Table 1.

Detection of ATP content

The ATP content of hiPSC-CMs was determined using the CellTiter-Glo 3D Cell Viability Assay (G9683, Promega, Madison, WI). Briefly, cardiac spheres were dissociated into single cells with 0.25% Trypsin-EDTA and replated into a 96-well plate (Corning, 3610) with 50,000 cells per well in 100 µL maturation medium that equilibrated to room temperature. CellTiter-Glo 3D reagent completely equilibrated to room temperature was added to the maturation medium (1:1) in each well containing cells followed by a shaking for 10 min at room temperature. After the incubation, luminescence was measured using the TopCount NXT Microplate Luminescence Counter (PerkinElmer) and TopCount NXT Software (integration time of 1 second per well). The luciferase reaction for this assay results in cell lysis and generation of a luminescent signal proportional to the amount of ATP present, and the amount of ATP is directly proportional to the number of viable cells present in culture under normal culture condition.

Mitochondrial membrane potential measurement

The mitochondrial membrane potential ($\Delta\Psi m$) was analyzed using the fluorescent dye tetramethylrhodamine, ethyl ester (TMRM; Thermo Fisher Scientific, Waltham, MA). After two weeks treatment with AMPK activators, cardiac spheres were dissociated using 0.25% trypsin-EDTA and plated onto a Matrigel-coated 96-well culture plate at a density of $2x10^4$ cells/well. CMs were maintained in the maturation medium for 24 h to allow cells to recover spontaneous beating. CMs were then labeled with 100 nM TMRM and Hoechst that were diluted in warm 0.2% BSA/PBS for 15 mins at 37°C in a 5% CO_2 incubator. After incubation, labeling solution was replaced with warm 0.2% BSA/PBS and immediately analyzed by ArrayScan. Data were expressed as the mean fluorescence intensity.

Quantification of mitochondrial DNA content

Cardiac spheres were dissociated with 0.25% trypsin/EDTA and total genomic DNA (gDNA) was isolated using QIAamp DNA Mini Kit (Qiagen, Venlo, Netherlands). Following determination of gDNA concentration using a UV-Vis spectrophotometer (NanoDrop, Thermo Fisher Scientific, Waltham, MA), samples were diluted to yield equal amounts of gDNA. Real-time PCR amplification was performed in an ABI Prism 7500 Real-Time PCR System (Applied BioSystems, Foster City, CA) for nuclear DNA (nDNA), succinate dehydrogenase subunit A [3] (*SDHA:* Fw-*TCTCCAGTGGCCAACAGTGTT;* Rw-*GCCCTCTTGTTCCCATCAAC*) and lipoprotein lipase [4] (*LPL:* Fw-*CGAGTCGTCTTTCTCCTGAT;* Rw-*TTCTGGATTCCAATGCTTCGA*), and mitochondrial DNA (mtDNA), NADH dehydrogenase subunit I [4] (*ND1:* Fw-*CCCTAAAACCCGCCACATCT;* Rw-*GAGCGATGGTGAGAGCTAAGGT*) and mitochondrial cytochrome oxidase II [3] (*mt-CO_2:* Fw-*CGATCCCTCCCTTAC;* Rw-*GAGAGGGGAGAGCAAT*). The mtDNA was normalized to nDNA.

Measurement of sarcomere length, cell area, cell perimeter and length/width ratio

Cardiac spheres at day 28 were dissociated with 0.25% trypsin/EDTA and plated on Matrigel-coated Lab-Tek chamber slides (Thermo/Nunc) for 4 days culture until cells completely recovered and spread out. Cells were then fixed and stained with NKX2-5 & a-actinin antibodies and Hoechst following the protocol described in section 'Immunocytochemical analysis'. The stained cells were imaged using an inverted microscope (Axio Vert.A1, Zeiss, Oberkochen, Germany) at 40X magnification and sarcomere length, cell area, cell perimeter and length/width ratio were captured using NIH ImageJ software. Statistical analysis was performed from measurements of more than

100 isolated CMs for each condition. Ten consecutive sarcomeres were measured and the sarcomere length was quantified as the measurement divided by 10.

Fatty acid uptake assay

After 7- and 14-days treatment, the cardiac spheres were dissociated into single cells and replated into Matrigel-coated 96-well plate at a density of 50000 cells per well in CM maturation medium supplemented with ROCK inhibitor. The next day, the medium was refreshed with CM maturation medium supplemented with AMPK activators. Cells were allowed to recover for one more day and fatty acid uptake assay was performed using the Free Fatty Acid Uptake Assay Kit (ab176768, fluorometric; Abcam, Boston, MA) following the manufacturer's instructions. After adding the fatty acid dye-loading solution into each well, the fluorescence signal was measured at 10 mins interval for 90 mins with a fluorescence microplate reader at Ex/Em = 485/515 nm using a bottom read mode. The cells were then trypsinized and counted with Typan Blue. The fluorescence intensity was normalized to 100000 cells.

Glucose uptake assay

After 7- and 14-days treatment, the cardiac spheres were dissociated into single cells and replated into Matrigel-coated 96-well plates at a density of 100000 cells per well in CM maturation medium supplemented with ROCK inhibitor. Four plates were prepared for this assay. Each plate contained all the treatments. In the next day, culture medium was replaced with CM maturation medium supplemented with AMPK activators and glucose uptake assay was performed on the CM maturation medium using the Glucose Uptake Assay Kit (ab65333, fluorometric; Abcam, Boston, MA) following the manufacturer's instructions (Day 0). The fluorescence signals were measured on a microplate reader at Ex/Em = 538/587 nm using a bottom read mode. Starting Day 1 to Day 4, the assay was performed on one plate per day followed by a cell counting for all treatments.

Calcium imaging

Cardiac spheres were dissociated with 0.25% Trypsin/EDTA and replated onto Matrigel (1:30)-coated 25x25x1 mm glass coverslips at the density of 50,000 cells per coverslip and cultured in maturation medium for an additional 4-5 days. For calcium imaging, cells were incubated with 5 μM Fluo-4 AM dye (F14201; Thermo Fisher Scientific, Waltham, MA) for 15 min at 37℃ in Tyrode's solution (148 mM NaCl, 4 mM KCl, 0.5 mM $MgCl_2 \cdot 6H_2O$, 0.3 mM $NaPH_2O_4 \cdot H_2O$, 5 mM HEPES, 1.8 mM $CaCl_2 \cdot H_2O$, and 10 mM D-glucose, pH adjusted to 7.4 with NaOH) supplemented with Pluronic™ F-127 (1:500 dilution; P3000MP; Thermo Fisher Scientific, Waltham, MA) and washed once with Tyrode's solution. Cells were subsequently transferred to a temperature-controlled microscope chamber coupled to an inverted laser confocal scanning microscope (Olympus FV1000, Olympus, Tokyo, Japan) equipped with FluoView software (Olympus, Tokyo, Japan) and cells were continuously perfused at 37 ℃ with Tyrode's solution. The intracellular calcium fluorescence signals were acquired at 40X magnification when cells were stimulated at 0.5 Hz. The recordings were analyzed with ClampFit 10.6 software (Molecular Devices, San Jose, CA).

Measurement of action potential using FluoVolt™

Cardiac spheres were dissociated with 0.25% Trypsin/EDTA and replated onto Matrigel (1:30)-coated 25x25x1 mm glass coverslips at the density of 50,000 cells per coverslip and cultured in maturation medium for an additional 4-5 days. For FluoVolt™ imaging, cells were incubated with 1X FluoVolt™ membrane potential probe (F10488; Thermo Fisher Scientific, Waltham, MA) for 15 min at 37 °C in Tyrode's solution and washed once with Tyrode's solution. Cells were subsequently transferred to a temperature-controlled microscope chamber coupled to an inverted laser confocal scanning microscope (Olympus FV1000, Olympus, Tokyo, Japan) equipped with

FluoView software (Olympus, Tokyo, Japan) and cells were continuously perfused at 37 °C with Tyrode's solution. The intracellular FluoVolt^{TM} fluorescence signals were acquired at 40X magnification when cells were stimulated at 0.5 Hz. Data were analyzed with MATLAB software (MathWorks, Natick, MA). Electrophysiological parameters measured included: dF/dT; rise time to peak; normalized action potential amplitude; APD50, action potential duration at 50% of repolarization; APD80, action potential duration at 80% of repolarization.

Contractility analysis
Cardiac spheres were replated onto Matrigel-coated 6 well plates in maturation medium and maintained for one day before recording. Contracting spheres were video-recorded using a Leica DM IRBE inverted microscope (Leica, Germany) equipped with a controlled CO_2 and temperature chamber (5% CO_2, 37 °C). Cells were stimulated at 2 Hz and recorded at 10x magnification, a frame rate of 50 fps and a resolution of 1024x1024 pixels. Movie images were analyzed with MATLAB R2016b software (MathWorks, Natick, MA), using the open source optical flow software [5].

RNA Sequencing analysis
RNA was extracted from both hiPSC-CMs with and without AMPK activation using Aurum total RNA mini kit (Bio-Rad, Hercules, CA) according to manufacturer's instructions. Library preparation and sequencing were performed at the Novogene Co., LTD (Beijing, China). Reference genome and gene model annotation files were downloaded from genome website browser (NCBI/UCSC/Ensembl) directly. Indexes of the reference genome was built using STAR and paired-end clean reads were aligned to the reference genome using STAR (v2.5). HTSeq v0.6.1 was used to count the read numbers mapped of each gene. Differential expression analysis between two conditions/groups (two biological replicates per condition) was performed using the DESeq2 R package (1.34.0). The resulting P-values were adjusted using the Benjamini and Hochberg's approach for controlling the False Discovery Rate (FDR). Genes with an adjusted P-value < 0.05 found by DESeq2 were assigned as differentially expressed. Gene Ontology (GO) enrichment analysis of differentially expressed genes was done using clusterProfiler R package (4.2.2). GO terms with corrected P-value < 0.05 were considered significantly enriched by differential expressed genes.

Proteomics analysis
hiPSC-CMs with or without E10 treatment (n=3/group; 3-4x10^6 cells per sample) were lysed in the lysis buffer (50 mM HEPES pH = 7.4, 150 mM NaCl, 0.5% SDC, 10 units/mL benzonase, and 1 tablet/10 mL protease inhibitor) at 4 °C for 45 min. The protein concentration in the cell lysate was determined by the BCA assay, and protein amounts in all samples were then normalized based on their concentrations. Proteins were reduced with 5 mM DTT (56 °C, 30 min) and alkylated with 14 mM iodoacetamide (RT, 30 min in the dark). They were purified through the methanol-chloroform protein precipitation method. The isolated proteins were digested with trypsin in a buffer containing 50 mM HEPES pH 8.5, 1.6 M urea at 37 °C overnight. After digestion, peptides were purified using tC18 Sep-Pak cartridges. Tandem mass tag labeling and peptide fractionation, LC-MS/MS (liquid chromatography with tandem mass spectrometry) analysis, database search, data filtering, peptide quantification, and bioinformatic analysis were conducted as described previously [6]. Proteins were considered being up- or down-regulated when the abundance changed by >1.3-fold between two groups. Gene functional enrichment was performed with Metascape [7]. GO terms with $P < 0.05$ were considered to be significantly enriched among differentially expressed proteins.

High-resolution metabolomics

hiPSC-CMs treated with or without AMPK activation (n=3/group; 1-2 X 10^6 per sample) were lysed and extracted with ice cold acetonitrile and water (2:1 v/v). Cells extracts were analyzed as described previously [8-10]. Briefly, thawed extracts were incubated at 4 °C for 30 min, centrifuged at 16100 g for 10 min to remove protein and transferred to a refrigerated (4 °C) autosampler for analysis. Samples (n=6 per group) were analyzed using ultra-high-resolution mass spectrometry with hydrophilic interaction liquid chromatography (HILIC) [Accucore HILIC 100 x 2.1 mm columns]. Electrospray ionization was used in the positive ion mode on a Thermo Scientific Q-Exactive HF mass spectrometer (Thermo Fisher Scientific, Waltham, MA) operating with a resolution of 120000 and scan range of 85–1275 *m*/*z* (mass to charge) [10, 11]. Analyte separation for HILIC was performed with a Waters XBridge BEH Amide XP HILIC column (2.1 X 50 mm^2, 2.6 µm particle size) and gradient elution with mobile phases including water, acetonitrile and formic acid as described previously [12]. Raw data were extracted with apLCMS [13] and xMSanalyzer [14], pre-filtered and normalized as described previously [15]. Significant features (raw $P < 0.05$ by limma test) were further studied by pathway enrichment analysis using mummichog [16]. This approach protects against type 2 statistical error by including all features at $P < 0.05$ and protects against type 1 statistical error by permutation testing. Hierarchical cluster analysis and principal component analysis were used for untargeted comparison of the significant features associated with Cd (raw $P < 0.05$ by limma test) across all groups.

Cell viability assay
After pathological stimulation, cells in black wall 96-well plates (3603, Corning, NY) were subjected to cell viability assay. Cell viability was analyzed using the CellTiter-Blue Cell Viability Assay (G8081, Promega, Madison, WI), which is a fluorometric method for estimating the number of viable cells present in multiwell plates. It uses the indicator dye resazurin to measure the metabolic capacity of cells. Viable cells retain the ability to reduce resazurin into resorufin, which is highly fluorescent. Nonviable cells lose metabolic capacity, do not reduce the indicator dye, and thus do not generate a fluorescent signal. Briefly, 20 µl CellTiter-Blue reagent was added to 100 µl low-glucose culture medium (1:5 dilution) in each well. After the incubation for 2-4 h at 37°C, fluorescence was measured using the Gen5 3.03 Microplate Reader and Imager Software (Agilent Technologies, Santa Clara, CA) with an excitation wavelength of 530 nm and an emission wavelength of 590 nm.

Nile red staining
After pathological stimulation, cardiac spheres were dissociated and plated as single cells into Matrigel-coated standard or black wall 96-well plates at 20,000 cells per well. The next day, Cayman's Lipid Droplets Fluorescence Assay Kit (500001, Cayman Chemical, Ann Arbor, MI) was used to quantify the lipid droplets. Briefly, the cells were fixed with the Fixative Solution for 10 min and then stained with the Nile Red Staining Solution for 15 min. The mean fluorescence intensity of Nile Red staining was analyzed using high content imaging by ArrayScan.

Detection of cell apoptosis
After pathological stimulation, apoptosis of the cells in standard 96-well plates was detected using Caspase-Glo 3/7 Assay System (G8091, Promega, Madison, WI). Caspase-Glo 3/7 reagent was mixed with low-glucose culture medium at 1:1 ratio and added to each well with 100 µl per well. Blank reaction in the wells without cells was used to measure background luminescence associated with the plate and assay reagents. Luminescent signals were detected 1 hour after adding the Caspase-Glo 3/7 reagent using the TopCount NXT Microplate Luminescence Counter (PerkinElmer) and TopCount NXT Software.

Supplementary References

[1] C. Gentillon, D. Li, M. Duan, W.M. Yu, M.K. Preininger, R. Jha, A. Rampoldi, A. Saraf, G.C. Gibson, C.K. Qu, L.A. Brown, C. Xu, Targeting HIF-1alpha in combination with PPARalpha activation and postnatal factors promotes the metabolic maturation of human induced pluripotent stem cell-derived cardiomyocytes, J Mol Cell Cardiol 132 (2019) 120-135.

[2] R. Jha, Q. Wu, M. Singh, M.K. Preininger, P. Han, G. Ding, H.C. Cho, H. Jo, K.O. Maher, M.B. Wagner, C. Xu, Simulated Microgravity and 3D Culture Enhance Induction, Viability, Proliferation and Differentiation of Cardiac Progenitors from Human Pluripotent Stem Cells, Sci Rep 6 (2016) 30956.

[3] N. San Martin, A.M. Cervera, C. Cordova, D. Covarello, K.J. McCreath, B.G. Galvez, Mitochondria determine the differentiation potential of cardiac mesoangioblasts, Stem Cells 29(7) (2011) 1064-74.

[4] X. Yang, M. Rodriguez, L. Pabon, K.A. Fischer, H. Reinecke, M. Regnier, N.J. Sniadecki, H. Ruohola-Baker, C.E. Murry, Tri-iodo-l-thyronine promotes the maturation of human cardiomyocytes-derived from induced pluripotent stem cells, J Mol Cell Cardiol 72 (2014) 296-304.

[5] N. Huebsch, P. Loskill, M.A. Mandegar, N.C. Marks, A.S. Sheehan, Z. Ma, A. Mathur, T.N. Nguyen, J.C. Yoo, L.M. Judge, C.I. Spencer, A.C. Chukka, C.R. Russell, P.L. So, B.R. Conklin, K.E. Healy, Automated Video-Based Analysis of Contractility and Calcium Flux in Human-Induced Pluripotent Stem Cell-Derived Cardiomyocytes Cultured over Different Spatial Scales, Tissue Eng Part C Methods 21(5) (2015) 467-79.

[6] R. Liu, F. Sun, P. Forghani, L.C. Armand, A. Rampoldi, D. Li, R. Wu, C. Xu, Proteomic Profiling Reveals Roles of Stress Response, Ca(2+) Transient Dysregulation, and Novel Signaling Pathways in Alcohol-Induced Cardiotoxicity, Alcohol Clin Exp Res 44(11) (2020) 2187-2199.

[7] Y. Zhou, B. Zhou, L. Pache, M. Chang, A.H. Khodabakhshi, O. Tanaseichuk, C. Benner, S.K. Chanda, Metascape provides a biologist-oriented resource for the analysis of systems-level datasets, Nat Commun 10(1) (2019) 1523.

[8] J. Fernandes, J.D. Chandler, K.H. Liu, K. Uppal, Y.M. Go, D.P. Jones, Putrescine as indicator of manganese neurotoxicity: Dose-response study in human SH-SY5Y cells, Food Chem Toxicol 116(Pt B) (2018) 272-280.

[9] Y.M. Go, K. Uppal, D.I. Walker, V. Tran, L. Dury, F.H. Strobel, H. Baubichon-Cortay, K.D. Pennell, J.R. Roede, D.P. Jones, Mitochondrial metabolomics using high-resolution Fourier-transform mass spectrometry, Methods Mol Biol 1198 (2014) 43-73.

[10] K.H. Liu, D.I. Walker, K. Uppal, V. Tran, P. Rohrbeck, T.M. Mallon, D.P. Jones, High-Resolution Metabolomics Assessment of Military Personnel: Evaluating Analytical Strategies for Chemical Detection, J Occup Environ Med 58(8 Suppl 1) (2016) S53-61.

[11] Q.A. Soltow, F.H. Strobel, K.G. Mansfield, L. Wachtman, Y. Park, D.P. Jones, High-performance metabolic profiling with dual chromatography-Fourier-transform mass spectrometry (DC-FTMS) for study of the exposome, Metabolomics 9(1 Suppl) (2013) S132-S143.

[12] K.H. Liu, M. Nellis, K. Uppal, C. Ma, V. Tran, Y. Liang, D.I. Walker, D.P. Jones, Reference Standardization for Quantification and Harmonization of Large-Scale Metabolomics, Anal Chem 92(13) (2020) 8836-8844.

[13] T. Yu, Y. Park, J.M. Johnson, D.P. Jones, apLCMS--adaptive processing of high-resolution LC/MS data, Bioinformatics 25(15) (2009) 1930-6.

[14] K. Uppal, Q.A. Soltow, F.H. Strobel, W.S. Pittard, K.M. Gernert, T. Yu, D.P. Jones, xMSanalyzer: automated pipeline for improved feature detection and downstream analysis of large-scale, non-targeted metabolomics data, BMC Bioinformatics 14 (2013) 15.

[15] X. Hu, J.D. Chandler, S. Park, K. Liu, J. Fernandes, M. Orr, M.R. Smith, C. Ma, S.M. Kang, K. Uppal, D.P. Jones, Y.M. Go, Low-dose cadmium disrupts mitochondrial citric acid cycle and lipid metabolism in mouse lung, Free Radic Biol Med 131 (2019) 209-217.
[16] S. Li, Y. Park, S. Duraisingham, F.H. Strobel, N. Khan, Q.A. Soltow, D.P. Jones, B. Pulendran, Predicting network activity from high throughput metabolomics, PLoS Comput Biol 9(7) (2013) e1003123.

Supplementary Table 1. Antibodies

Type	Antibody target	Origin type	Supplier	Catalog number	Dilution
Primary	α-actinin	Mouse IgG1	Sigma	A7811	1:500 ICC; 1:800 Flow Cytometry
	NKX2-5	Rabbit IgG	Cell Signaling	8792S	1:1000
	TOM20	Mouse IgG2a	Santa Cruz	sc-17764	1:100 ICC; 1:200 Flow Cytometry
	Troponin I	Mouse IgG2b	Millipore	MAB1691	1:400
Secondary	Alexa Fluor 488, goat anti-mouse IgG1		Thermo Fisher	A21121	1:500
	Alexa Fluor 594, goat anti-mouse IgG2a		Thermo Fisher	A21135	1:500
	Alexa Fluor 594, goat anti-mouse IgG2b		Thermo Fisher	A21145	1:500
	Alexa Fluor 594, goat anti-rabbit IgG		Thermo Fisher	A11012	1:500

Supplementary Table 2. qRT-PCR Primers

Gene	Full name	Accession Code	Primer
PRKAA1	Protein kinase AMP-activated catalytic subunit alpha 1	NM_006251	CACATCAAGGCTCCGAATCT<br>ACCTTCGGCAAAGTGAAGG
PRKAA2	protein kinase AMP-activated catalytic subunit alpha 2	NM_006252	AACTGCCACTTTATGGCCTG<br>CGGGTGAAGATCGGACACTA
ADRB1	Adrenergic, beta-1-, receptor	NM_000684	ATCGAGACCCTGTGTGTCATT<br>GTAGAAGGAGACTACGGACGAG
GAPDH	Glyceraldehyde-3-phosphate dehydrogenase	NM_00125679 9	CTGGGCTACACTGAGCACC<br>AAGTGGTCGTTGAGGGCAATG

SLC2A1	Solute carrier family 2 (Facilitated glucose transporter), member 1	NM_006516	ACTCCTCGATCACCTTCTGG<br>ATGGAGCCCAGCAGCAA
SLC2A4	Solute carrier family 2 (Facilitated glucose transporter), member 4	NM_001042	AGCACCGCAGAGAACACAG<br>GTCGGGCTTCCAACAGATAG
PKM2	Pyruvate kinase M1/2	NM_002654	AAGGACCTGAGATCCGAACTG<br>GCGTTATCCAGCGTGATTTTGA
TBP	TATA-box binding protein	NM_003194	TGAGTTGCTCATACCGTGCTGCTA<br>CCCTCAAACCAACTTGTCAACAGC
LDHA	Lactate dehydrogenase A	NM_005566	TCCTTAGTGTTCCTTGCATTT<br>AAGTGTATCTGCACTCTTCTTC
GPD1	glycerol-3-phosphate dehydrogenase (NAD(+))	NM_005276	CCTTGTTCATGGCTGTGT<br>CACTCACATATGTTCTGGATGA
PLIN2	Perilipin 2	NM_001122	AATTCAGGATGCTCAGGATAAG<br>ACAGTGGGACTCATCAGTAT
LIPE (HSL)	Lipase E, hormone sensitive type	NM_005357	GATTCCCTCAAGAACCTGAC<br>GCATCCTCAGGTGGTAATAAG
CASP9	Caspase 9	NM_001229	TCGCTAATGCTGTTTCGG<br>CCCTGGCCTTATGATGTTT

Supplementary Table 3. Top 120 shared upregulated genes between E10-treated hiPSC-CMs and LV compared to DMSO-treated hiPSC-CMs.

Symbol	Gene name	Log_2FC (E10 vs. DMSO)	Log_2FC (LV vs. DMSO)
SV2B	synaptic vesicle glycoprotein 2B	3.217475702	4.579133423
CD274	CD274 molecule	2.22303859	5.371118037
ACSS1	acyl-CoA synthetase short chain family member 1	2.121414027	9.001837476
HPCAL4	hippocalcin like 4	1.9632831	2.354742585
VSIR	V-set immunoregulatory receptor	1.80701451	4.005860669
IFIT3	interferon induced protein with tetratricopeptide repeats 3	1.770141207	3.807859272
HS3ST1	heparan sulfate-glucosamine 3-sulfotransferase 1	1.687472135	2.091103887
HHATL	hedgehog acyltransferase like	1.609391546	5.961311283
C11orf21	chromosome 11 open reading frame 21	1.599925504	0.819065354
EPN3	epsin 3	1.59439206	2.421385664
RARRES3	retinoic acid receptor responder 3	1.584829494	2.069751074
BTN3A3	butyrophilin subfamily 3 member A3	1.584243259	3.714148716
ASB11	ankyrin repeat and SOCS box containing 11	1.526014811	2.890313988
KCNJ2	potassium voltage-gated channel subfamily J member 2	1.470906632	7.089319398
PSMB9	proteasome subunit beta 9	1.452901834	1.745720498
HLA-F	major histocompatibility complex, class I, F	1.422936476	3.199517888
NPY6R	neuropeptide Y receptor Y6 (pseudogene)	1.395126745	5.330519773
NMRK2	nicotinamide riboside kinase 2	1.390308687	6.275502737
VWC2	von Willebrand factor C domain containing 2	1.310147179	2.705042211
C2orf88	chromosome 2 open reading frame 88	1.309163031	5.698965657
UCP3	uncoupling protein 3	1.308024103	2.427083489
APOL6	apolipoprotein L6	1.303388102	1.190658943
FGL2	fibrinogen like 2	1.269980532	2.715342709
LPAR5	lysophosphatidic acid receptor 5	1.269679336	2.876981064
APOL3	apolipoprotein L3	1.255735449	4.61141855
PSMB8	proteasome subunit beta 8	1.228384706	1.143297484
SMIM5	small integral membrane protein 5	1.22067629	5.442852859
MT-TT	mitochondrially encoded tRNA threonine	1.218957707	3.091026419
LINC02517	long intergenic non-protein coding RNA 2517	1.162470221	1.479229919
RNF144B	ring finger protein 144B	1.155944898	4.04282495
COX6A2	cytochrome c oxidase subunit 6A2	1.138714626	6.498607362
HTRA3	HtrA serine peptidase 3	1.091266763	1.825747511
ABCG1	ATP binding cassette subfamily G member 1	1.090985847	1.597589242
ABCC9	ATP binding cassette subfamily C member 9	1.078179176	4.682824333
EPAS1	endothelial PAS domain protein 1	1.078151231	5.110019504
CMPK2	cytidine/uridine monophosphate kinase 2	1.072706308	1.857531885
SLC16A14	solute carrier family 16 member 14	1.070233982	3.123955481
CAVIN2	caveolae associated protein 2	1.065459191	6.88796068
TNNI3	troponin I3, cardiac type	1.062390883	8.860401275
HCN2	hyperpolarization activated cyclic nucleotide gated potassium and sodium channel 2	1.055664234	2.106891035

MT-TN	mitochondrially encoded tRNA asparagine	1.031930379	3.493881974
SCN1B	sodium voltage-gated channel beta subunit 1	1.025446095	3.032987243
SGK1	serum/glucocorticoid regulated kinase 1	1.02360783	5.146094945
PFKFB2	6-phosphofructo-2-kinase/fructose-2,6-biphosphatase 2	1.020940091	3.269576051
LPL	lipoprotein lipase	1.020058781	9.440800847
COQ10A	coenzyme Q10A	0.996405719	2.690538396
ASAH1	N-acylsphingosine amidohydrolase 1	0.980181573	2.682115088
GRK5	G protein-coupled receptor kinase 5	0.979841171	2.399076986
FAM129A	family with sequence similarity 129 member A	0.978393181	8.307951162
B3GNT8	UDP-GlcNAc:betaGal beta-1,3-N-acetylglucosaminyltransferase 8	0.976286096	1.649260115
RENBP	renin binding protein	0.973587571	1.074408262
LRRC39	leucine rich repeat containing 39	0.966733618	3.430732008
UCP2	uncoupling protein 2	0.955535967	2.348263328
ELAC1	elaC ribonuclease Z 1	0.95548826	1.551084193
ABLIM3	actin binding LIM protein family member 3	0.950710798	4.447351918
ANKRD44	ankyrin repeat domain 44	0.938252378	1.728008666
GPNMB	glycoprotein nmb	0.924271322	8.839663905
DUSP6	dual specificity phosphatase 6	0.874985599	1.664485344
KBTBD13	kelch repeat and BTB domain containing 13	0.867169268	2.063744243
FRK	fyn related Src family tyrosine kinase	0.843423002	1.736854591
UST	uronyl 2-sulfotransferase	0.842261014	2.105673252
SEMA3C	semaphorin 3C	0.818229684	2.473994478
NEU3	neuraminidase 3	0.811291908	1.231327026
PPP1R12B	protein phosphatase 1 regulatory subunit 12B	0.802488655	2.803379073
CPVL	carboxypeptidase, vitellogenic like	0.80220239	2.146231421
COX7B	cytochrome c oxidase subunit 7B	0.797611174	2.682768205
CKMT2	creatine kinase, mitochondrial 2	0.785844629	4.535576717
SEC11C	SEC11 homolog C, signal peptidase complex subunit	0.775103792	0.699868092
ADRB1	adrenoceptor beta 1	0.771019825	3.802709217
MIR22HG	MIR22 host gene	0.765570422	2.931879732
MYPN	myopalladin	0.753404895	3.662061075
TAP1	transporter 1, ATP binding cassette subfamily B member	0.753038351	0.687562051
MT-CYB	mitochondrially encoded cytochrome b	0.746845799	3.710447084
MT-ND2	mitochondrially encoded NADH:ubiquinone oxidoreductase core subunit 2	0.740961803	3.194276937
IFI35	interferon induced protein 35	0.734910921	1.162198118
MTND2P28	MT-ND2 pseudogene 28	0.733474427	3.951849465
ARSG	arylsulfatase G	0.730567142	0.617823949
MT-CO3	mitochondrially encoded cytochrome c oxidase III	0.724255705	3.549908708
APOL2	apolipoprotein L2	0.723232879	0.467689909
U91328.1	novel transcript	0.722355182	2.17706158
MYH14	myosin heavy chain 14	0.721589553	3.906668992
INSYN1	inhibitory synaptic factor 1	0.719853858	3.020511116
SLC46A3	solute carrier family 46 member 3	0.714761177	0.95495195

ABCB4	ATP binding cassette subfamily B member 4	0.713237903	2.154261016
B2M	beta-2-microglobulin	0.711819285	1.63917741
SMCO1	single-pass membrane protein with coiled-coil domains 1	0.711480688	3.549741737
PDE7A	phosphodiesterase 7A	0.69078983	3.070706069
CGRRF1	cell growth regulator with ring finger domain 1	0.689749663	1.196632468
GCOM1	GRINL1A complex locus 1	0.677008402	1.058569942
MIR133A1HG	MIR133A1 host gene	0.672590254	3.210620469
FGF7	fibroblast growth factor 7	0.671156778	1.288279523
C5orf56	chromosome 5 open reading frame 56	0.67046758	0.873478124
TMCO4	transmembrane and coiled-coil domains 4	0.666508131	1.519935608
MT-ND3	mitochondrially encoded NADH:ubiquinone oxidoreductase core subunit 3	0.659989967	3.408195305
TTC39A	tetratricopeptide repeat domain 39A	0.655666444	0.869804551
PARP10	poly(ADP-ribose) polymerase family member 10	0.652828866	1.950160787
AC116351.1	uncharacterized LOC100506688	0.651727507	2.215071539
HERPUD1	homocysteine inducible ER protein with ubiquitin like domain 1	0.651108897	1.366556245
PTPRB	protein tyrosine phosphatase, receptor type B	0.639799548	4.620936977
SLC2A4	solute carrier family 2 member 4	0.636945154	2.100753274
NIPAL2	NIPA like domain containing 2	0.635535294	1.287418656
IL1R1	interleukin 1 receptor type 1	0.630529009	1.834900904
PIP5K1B	phosphatidylinositol-4-phosphate 5-kinase type 1 beta	0.623065503	2.871698295
GPRC5B	G protein-coupled receptor class C group 5 member B	0.615037843	2.45271109
C1QTNF1	C1q and TNF related 1	0.607682739	1.111353915
MT-ND1	mitochondrially encoded NADH:ubiquinone oxidoreductase core subunit 1	0.607327434	3.839297458
PLN	phospholamban	0.601068139	0.463387426
ALDH5A1	aldehyde dehydrogenase 5 family member A1	0.593368435	2.17868705
LACTB2	lactamase beta 2	0.592575088	1.057856656
SLC40A1	solute carrier family 40 member 1	0.592074414	2.154571663
MIPEP	mitochondrial intermediate peptidase	0.589723401	1.016664642
COMMD8	COMM domain containing 8	0.589510803	1.706057862
MYOZ2	myozenin 2	0.589138899	3.780824299
PDE1C	phosphodiesterase 1C	0.584486192	4.071020079
MT-CO2	mitochondrially encoded cytochrome c oxidase II	0.58200089	2.641251942
GPAT3	glycerol-3-phosphate acyltransferase 3	0.580714222	1.169740038
MT-CO1	mitochondrially encoded cytochrome c oxidase I	0.577759378	3.097655168
CLIP4	CAP-Gly domain containing linker protein family member 4	0.576215927	1.211668398
RPS6KL1	ribosomal protein S6 kinase like 1 [Source:	0.575844743	1.053001747
ITSN2	intersectin 2	0.573612215	0.951512991

Supplementary Table 4. Top 40 up-regulated proteins in E10-treated hiPSC-CMs

ID	Symbol	Full name	Fold change	*p*-value
P17540	CKMT2	creatine kinase, mitochondrial 2	2.054	1.532E-05
P14672	SLC2A4	solute carrier family 2 member 4	1.831	1.429E-04
P51606	RENBP	renin binding protein	1.800	4.100E-04
P05413	FABP3	fatty acid binding protein 3	1.773	3.442E-02
Q9Y5U8	MPC1	mitochondrial pyruvate carrier 1	1.715	8.339E-05
Q9BU61	NDUFAF3	NADH:ubiquinone oxidoreductase complex assembly factor 3	1.670	3.944E-04
P30405	PPIF	peptidylprolyl isomerase F	1.664	6.347E-06
P50213	IDH3A	isocitrate dehydrogenase (NAD(+)) 3 catalytic subunit alpha	1.658	3.640E-05
H0YIC4	CS	citrate synthase	1.612	4.318E-04
O43837	IDH3B	isocitrate dehydrogenase (NAD(+)) 3 non-catalytic subunit beta	1.593	8.901E-05
P61604	HSPE1	heat shock protein family E (Hsp10) member 1	1.581	1.616E-05
O15440	ABCC5	ATP binding cassette subfamily C member 5	1.575	4.599E-02
P02144	MB	myoglobin	1.571	1.185E-02
Q9Y2Q3	GSTK1	glutathione S-transferase kappa 1	1.567	2.671E-05
Q9UI09	NDUFA12	NADH:ubiquinone oxidoreductase subunit A12	1.566	1.112E-02
Q9H6R3	ACSS3	acyl-CoA synthetase short chain family member 3	1.563	3.729E-04
P51553	IDH3G	isocitrate dehydrogenase (NAD(+)) 3 non-catalytic subunit gamma	1.558	8.554E-06
Q8N4T8	CBR4	carbonyl reductase 4	1.553	3.583E-04
Q9C099	LRRCC1	leucine rich repeat and coiled-coil centrosomal protein 1	1.550	3.638E-04
P14406	COX7A2	cytochrome c oxidase subunit 7A2	1.544	1.171E-05
Q6ZVF9	GPRIN3	GPRIN family member 3	1.543	1.410E-03
O00483	NDUFA4	NDUFA4 mitochondrial complex associated	1.542	4.261E-04
Q9H019	MTFR1L	mitochondrial fission regulator 1 like	1.539	1.283E-05
P23368	ME2	malic enzyme 2	1.534	2.630E-03
O14949	UQCRQ	ubiquinol-cytochrome c reductase complex III subunit VII	1.531	2.488E-05
O95563	MPC2	mitochondrial pyruvate carrier 2	1.528	2.487E-05

Q7Z3D6	C14orf159	D-glutamate cyclase	1.523	7.983E-03
O60783	MRPS14	mitochondrial ribosomal protein S14	1.523	8.624E-04
Q5JTJ3	COA6	cytochrome c oxidase assembly factor 6	1.520	7.172E-03
Q96EY8	MMAB	metabolism of cobalamin associated B	1.517	5.518E-04
P05166	PCCB	propionyl-CoA carboxylase subunit beta	1.510	6.538E-04
P14927	UQCRB	ubiquinol-cytochrome c reductase binding protein	1.505	3.239E-07
P11310	ACADM	acyl-CoA dehydrogenase medium chain	1.504	1.286E-04
O14862	AIM2	absent in melanoma 2	1.504	2.515E-03
O75306	NDUFS2	NADH:ubiquinone oxidoreductase core subunit S2	1.504	3.232E-05
Q5T653	MRPL2	mitochondrial ribosomal protein L2	1.502	3.360E-02
Q9H2W6	MRPL46	mitochondrial ribosomal protein L46	1.501	6.882E-05
Q6NUK1	SLC25A24	solute carrier family 25 member 24	1.500	3.483E-03
P04179	SOD2	superoxide dismutase 2	1.500	2.696E-05
Q16795	NDUFA9	NADH:ubiquinone oxidoreductase subunit A9	1.495	1.377E-04

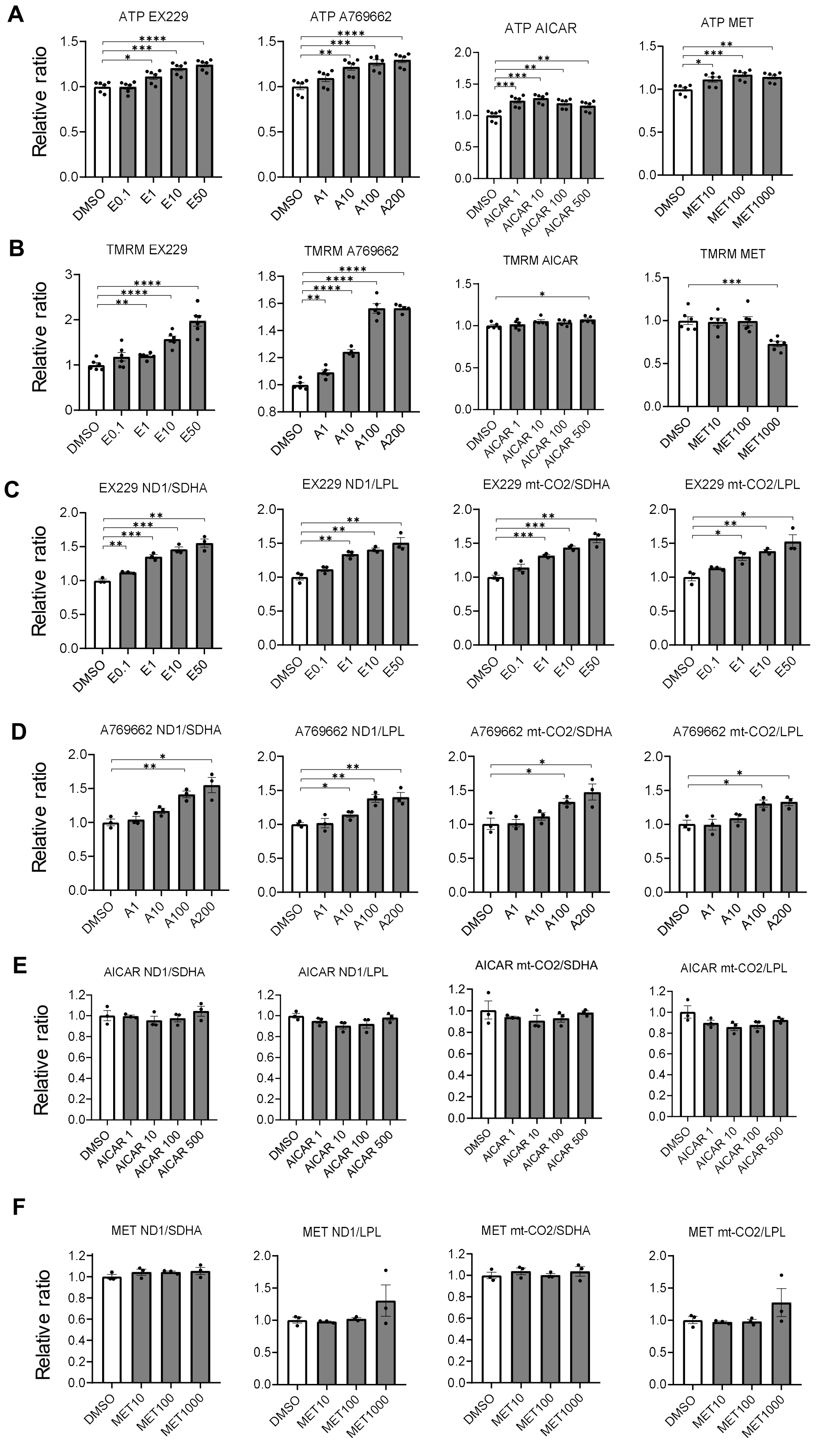
A
ATP EX229
ATP A769662
ATP AICAR
ATP MET
B
TMRM EX229
TMRM A769662
TMRM AICAR
TMRM MET
C
EX229 ND1/SDHA
EX229 ND1/LPL
EX229 mt-CO2/SDHA
EX229 mt-CO2/LPL
D
A769662 ND1/SDHA
A769662 ND1/LPL
A769662 mt-CO2/SDHA
A769662 mt-CO2/LPL
E
AICAR ND1/SDHA
AICAR ND1/LPL
AICAR mt-CO2/SDHA
AICAR mt-CO2/LPL
F
MET ND1/SDHA
MET ND1/LPL
MET mt-CO2/SDHA
MET mt-CO2/LPL
Relative ratio
DMSO
E0.1
E1
E10
E50
A1
A10
A100
A200
AICAR 1
AICAR 10
AICAR 100
AICAR 500
MET10
MET100
MET1000

Supplementary Figure 1. Optimal dose screening for AMPK activators in IMR90-derived cardiomyocytes. (A) Relative ATP content (n=6 cultures); (B) Relative TMRM determined using ArrayScan (n=5-6 cultures); and mtDNA/nDNA determined by qPCR for (C) EX229 (D) A-769662 (E) AICAR (F) MET (n=3 cultures). Data are represented as mean ± SEM. *P < 0.05, **P < 0.01, ***P < 0.001, ****P < 0.0001 by one-way ANOVA. DMSO, dimethyl sulfoxide; LPL, lipoprotein lipase; MET, metformin; mt-CO2, mitochondrially encoded cytochrome c oxidase II; mtDNA, mitochondrial DNA; ND1, mitochondrially encoded NADH dehydrogenase 1; nDNA, nuclear DNA; SDHA, succinate dehydrogenase complex flavoprotein subunit A; TMRM, tetramethylrhodamine, methyl ester.

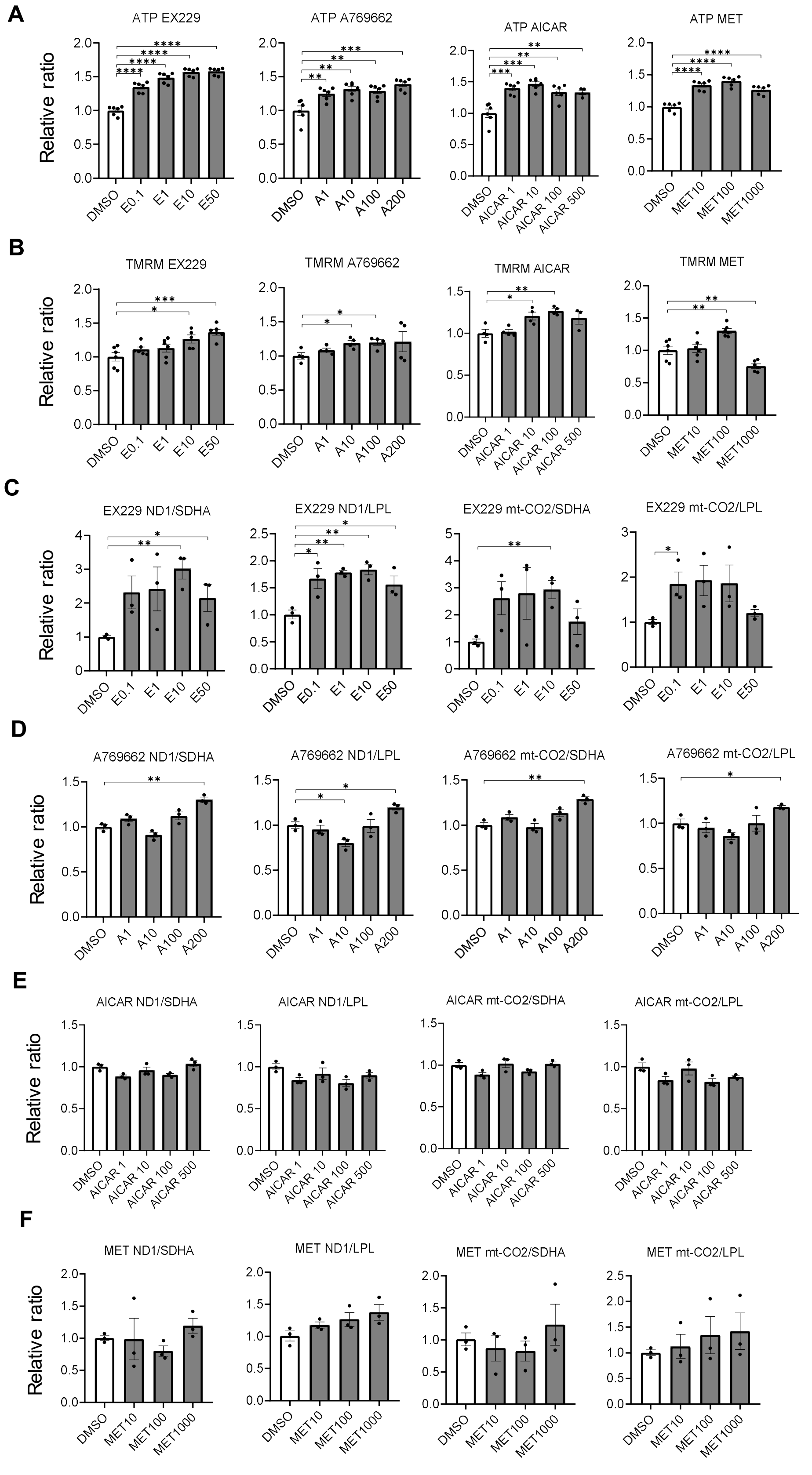
A
ATP EX229
ATP A769662
ATP AICAR
ATP MET
B
TMRM EX229
TMRM A769662
TMRM AICAR
TMRM MET
C
EX229 ND1/SDHA
EX229 ND1/LPL
EX229 mt-CO2/SDHA
EX229 mt-CO2/LPL
D
A769662 ND1/SDHA
A769662 ND1/LPL
A769662 mt-CO2/SDHA
A769662 mt-CO2/LPL
E
AICAR ND1/SDHA
AICAR ND1/LPL
AICAR mt-CO2/SDHA
AICAR mt-CO2/LPL
F
MET ND1/SDHA
MET ND1/LPL
MET mt-CO2/SDHA
MET mt-CO2/LPL
Relative ratio
DMSO
E0.1
E1
E10
E50
A1
A10
A100
A200
AICAR 1
AICAR 10
AICAR 100
AICAR 500
MET10
MET100
MET1000

Supplementary Figure 2. Optimal dose screening for AMPK activators in SCVI273-derived cardiomyocytes. (A) Relative ATP content (n=6 cultures); (B) Relative TMRM determined using ArrayScan (n=6 for EX229 and MET; n=4 for A769662 and AICAR); and mtDNA/nDNA determined by qPCR for (C) EX229 (D) A769662 (E) AICAR (F) MET (n=3 cultures). Data are represented as mean ± SEM. *P < 0.05, **P < 0.01, ***P < 0.001, ****P < 0.0001 by one-way ANOVA. DMSO, dimethyl sulfoxide; LPL, lipoprotein lipase; MET, metformin; mt-CO2, mitochondrially encoded cytochrome c oxidase II; mtDNA, mitochondrial DNA; ND1, mitochondrially encoded NADH dehydrogenase 1; nDNA, nuclear DNA; SDHA, succinate dehydrogenase complex flavoprotein subunit A; TMRM, tetramethylrhodamine, methyl ester.

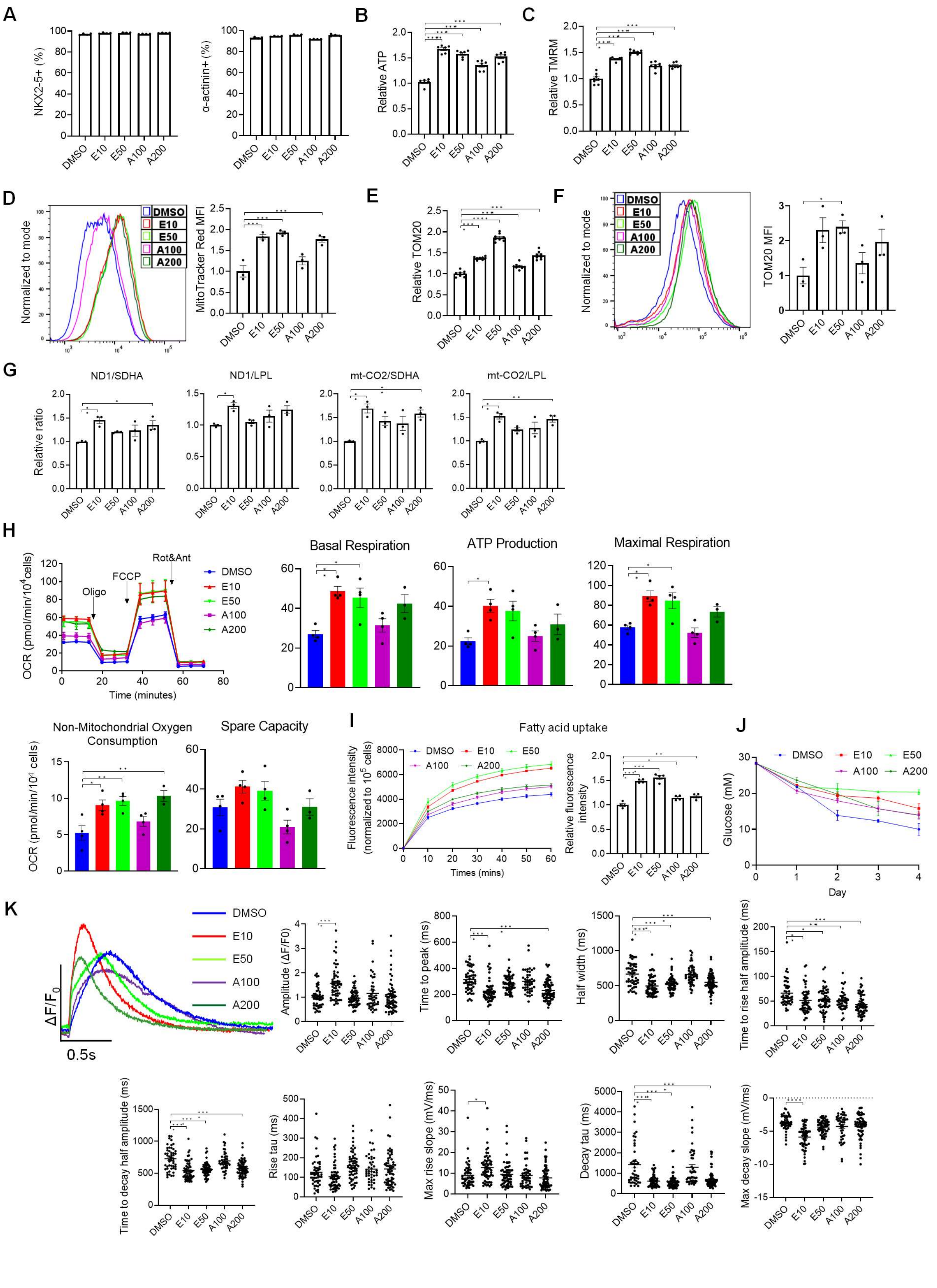
A
NKX2-5+ (%)
α-actinin+ (%)
B
Relative ATP
C
Relative TMRM
D
Normalized to mode
MitoTracker Red MFI
E
Relative TOM20
F
TOM20 MFI
G
ND1/SDHA
ND1/LPL
mt-CO2/SDHA
mt-CO2/LPL
Relative ratio
H
OCR (pmol/min/10^4 cells)
Oligo
FCCP
Rot&Ant
Time (minutes)
Basal Respiration
ATP Production
Maximal Respiration
Non-Mitochondrial Oxygen Consumption
Spare Capacity
I
Fatty acid uptake
Fluorescence intensity (normalized to 10^5 cells)
Times (mins)
Relative fluorescence intensity
J
Glucose (mM)
Day
K
ΔF/F0
0.5s
Amplitude (ΔF/F0)
Time to peak (ms)
Half width (ms)
Time to rise half amplitude (ms)
Time to decay half amplitude (ms)
Rise tau (ms)
Max rise slope (mV/ms)
Decay tau (ms)
Max decay slope (mV/ms)
DMSO
E10
E50
A100
A200

Supplementary Figure 3. AMPK activation for 14 days improves mitochondrial maturation. (A) Quantitative analysis of NKX2-5 and α-actinin by high-content imaging using ArrayScan on day 28 (n=4 cultures). (B) ATP content (n=5). (C) Quantitative analysis of TMRM by high-content imaging using ArrayScan (n=8 cultures). (D) Quantitative analysis of TOM20 by high-content imaging using ArrayScan (n=8). (E) Representative histograms of MitoTracker Red fluorescence intensity from flow cytometry analysis and the relative fluorescence intensity of AMPK activated and control hiPSC-CMs (n=3 cultures). (F) Representative histograms of TOM20 fluorescence intensity from flow cytometry analysis and the relative fluorescence intensity of AMPK activated and control hiPSC-CMs (n=3 cultures). (G) Mitochondrial content was measured as density of mtDNA to nDNA using qPCR. Bar graphs show ratios of ND1/LPL, ND1/SDHA, mt-CO2/LPL, and mt-CO2/SDHA (n=3 cultures). (H) Left: Representative traces showing the OCR of hiPSC-CMs following sequential addition of Oligo (2 μM), FCCP (1 μM), and Rot/Ant (0.5 μM). Right: Quantification of basal respiration, maximal respiration, spare respiratory capacity, non-mitochondrial respiration and ATP production. All measurements were normalized to cell counts (n=4 cultures). (I) Fatty acid uptake measured in maturation medium (n=4 cultures). (J) Glucose concentration measured in maturation medium over 4-day culture period (n=4 cultures). (K) Representative traces and quantification of calcium transients. Data are represented as mean ± SEM. *P < 0.05, **P < 0.01, ***P < 0.001, ****P < 0.0001 by one-way ANOVA. A100, A-769662 at 100 μM; A200, A-769662 at 200 μM; DMSO, dimethyl sulfoxide; E10, EX229 at 10 μM; E50, EX229 at 50 μM; FCCP, carbonyl cyanide p-(trifluoromethoxy) phenylhydrazone; LPL, lipoprotein lipase; MFI, mean fluorescence intensity; mt-CO2, mitochondrially encoded cytochrome c oxidase II; mtDNA, mitochondrial DNA; ND1, mitochondrially encoded NADH dehydrogenase 1; nDNA, nuclear DNA; NKX2-5, NK2 homeobox 5; OCR, oxygen consumption rate; Oligo, oligomycin; Rot/Ant, rotenone/antimycin A; SDHA, succinate dehydrogenase complex flavoprotein subunit A; TMRM, tetramethylrhodamine, methyl ester.

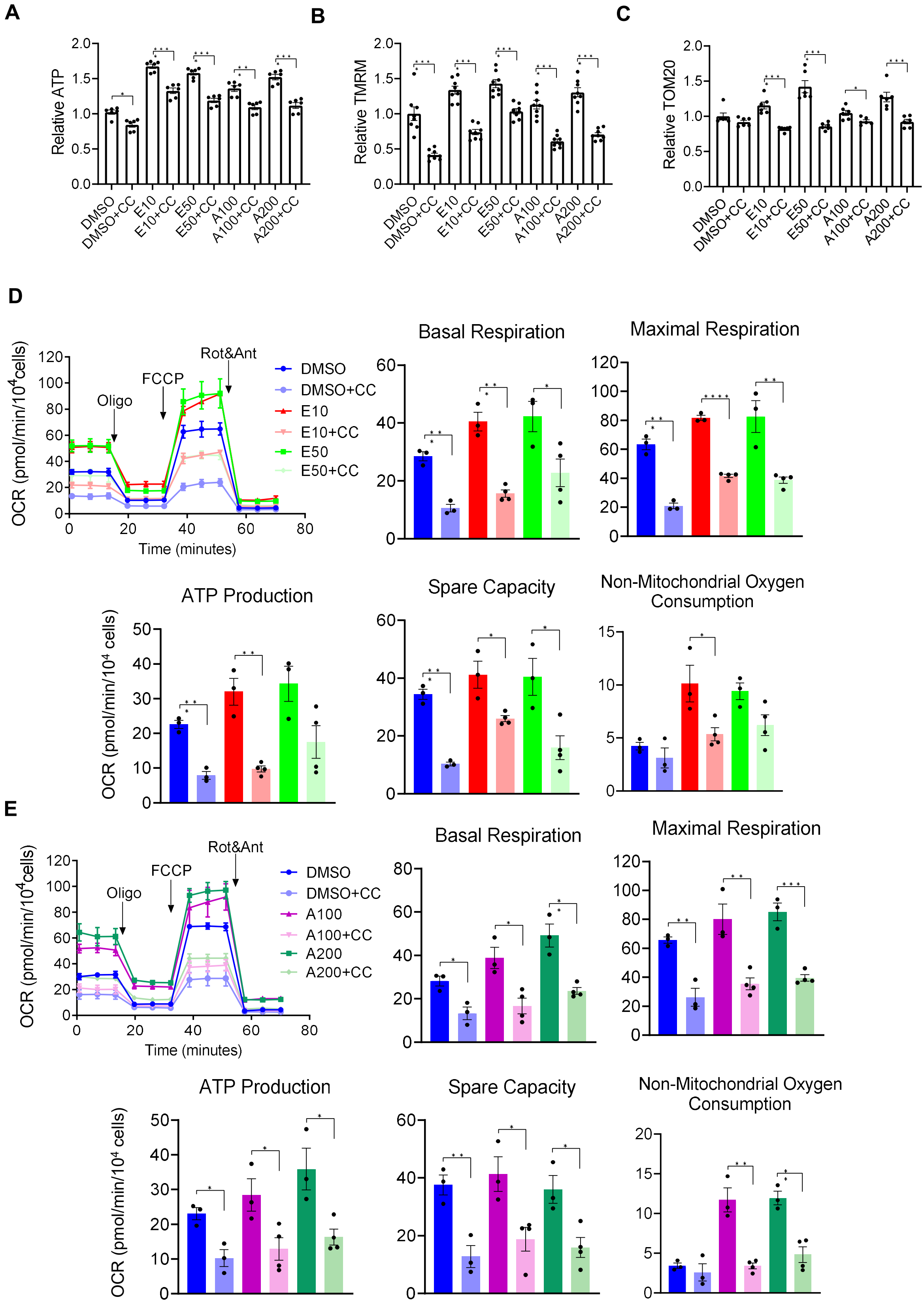
A
Relative ATP
DMSO
DMSO+CC
E10
E10+CC
E50
E50+CC
A100
A100+CC
A200
A200+CC
B
Relative TMRM
C
Relative TOM20
D
OCR (pmol/min/10^4cells)
Oligo
FCCP
Rot&Ant
Time (minutes)
DMSO
DMSO+CC
E10
E10+CC
E50
E50+CC
Basal Respiration
Maximal Respiration
ATP Production
OCR (pmol/min/10^4 cells)
Spare Capacity
Non-Mitochondrial Oxygen Consumption
E
OCR (pmol/min/10^4cells)
Oligo
FCCP
Rot&Ant
Time (minutes)
DMSO
DMSO+CC
A100
A100+CC
A200
A200+CC
Basal Respiration
Maximal Respiration
ATP Production
OCR (pmol/min/10^4 cells)
Spare Capacity
Non-Mitochondrial Oxygen Consumption

Supplementary Figure 4. AMPK knockdown for 14 days using Compound C inhibits the metabolic maturation of hiPSC-CMs. Measurements of (A) Relative ATP content (B) Relative TMRM indicating mitochondrial membrane potential (C) Relative TOM20 indicating mitochondrial content (D) The effect of EX229 on mitochondrial function was abolished with Compound C. Representative traces showing the OCR of hiPSC-CMs following sequential addition of Oligo (2 μM), FCCP (1 μM), and Rot/Ant (0.5 μM). Quantification of basal respiration, maximal respiration, ATP production, spare respiratory capacity, and non-mitochondrial respiration. All measurements were normalized to cell counts and presented as mean ± SEM (n=4 cultures). (E) The effect of A-769662 on mitochondrial function was abolished with Compound C. Representative traces showing the OCR of hiPSC-CMs following sequential addition of Oligo (2 μM), FCCP (1 μM), and Rot/Ant (0.5 μM). Quantification of basal respiration, maximal respiration, ATP production, spare respiratory capacity, and non-mitochondrial respiration. All measurements were normalized to cell counts and presented as mean ± SEM (n=4 cultures). A100, A-769662 at 100 μM; A200, A-769662 at 200 μM; DMSO, dimethyl sulfoxide; E10, EX229 at 10 μM; E50, EX229 at 50 μM; FCCP, carbonyl cyanide p-(trifluoromethoxy) phenylhydrazone; OCR, oxygen consumption rate; Oligo, oligomycin; Rot/Ant, rotenone/antimycin A; TMRM, tetramethylrhodamine, methyl ester.

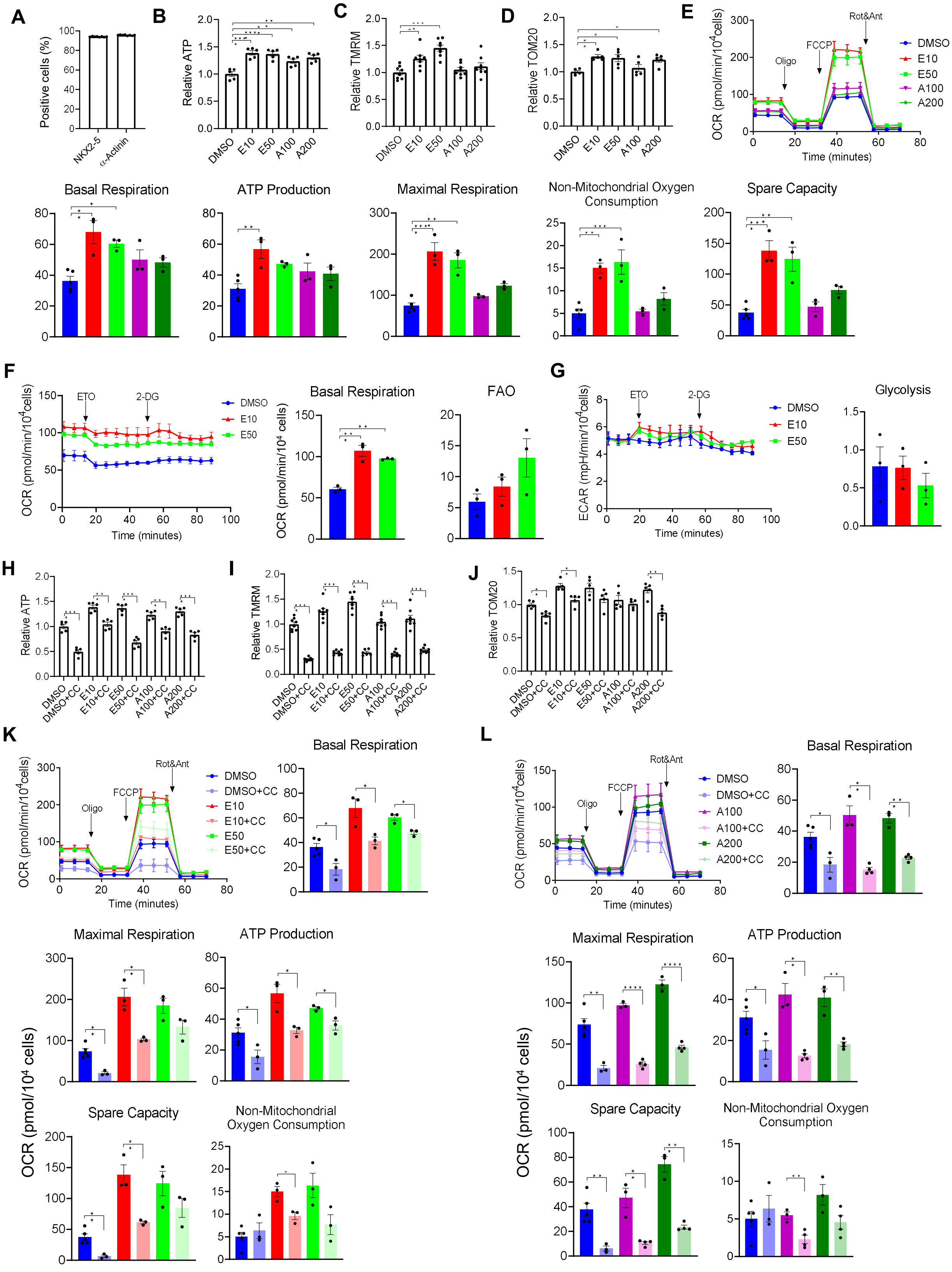
A
Positive cells (%)
NKX2-5
α-Actinin
B
Relative ATP
DMSO
E10
E50
A100
A200
C
Relative TMRM
D
Relative TOM20
E
OCR (pmol/min/10^4cells)
Oligo
FCCP
Rot&Ant
Time (minutes)
Basal Respiration
ATP Production
Maximal Respiration
Non-Mitochondrial Oxygen Consumption
Spare Capacity
F
ETO
2-DG
FAO
G
ECAR (mpH/min/10^4cells)
Glycolysis
H
DMSO+CC
E10+CC
E50+CC
A100+CC
A200+CC
I
J
K
L
OCR (pmol/10^4 cells)

Supplementary Figure 5. AMPK activation for 7 days improves metabolic maturation of SCVI273 derived cardiomyocytes. (A) Quantitative analysis of NKX2-5 and α-actinin positivity by high-content imaging using ArrayScan at day 21 (n=8 cultures). (B) ATP content. Data are presented as mean ± SEM (n=5). (C) Quantitative analysis of TMRM by high-content imaging using ArrayScan (n=8 cultures). (D) Quantitative analysis of TOM20 by high-content imaging using ArrayScan (n=5 cultures). (E) Representative traces of real-time measurement of OCR and quantification of mitochondrial functional parameters including basal respiration, maximal respiration, ATP production, non-mitochondrial respiration, and spare respiratory capacity. All measurements were normalized to cell counts (n=3 cultures). (F) Representative traces of real-time measurement of OCR and quantification of fatty acid oxidation (the amount of OCR derived from fatty acid oxidation). (G) Representative traces of real-time measurement of ECAR and quantification of glycolysis. Data were normalized to cell counts (n=3 cultures). AMPK knockdown using Compound C inhibits the metabolic maturation. Measurements of (H) Relative ATP content (I) Relative TMRM indicating mitochondrial membrane potential (J) Relative TOM20 indicating mitochondrial content (K) The effect of EX229 on mitochondrial function was abolished with Compound C. Representative traces showing the OCR of hiPSC-CMs following sequential addition of Oligo (2 μM), FCCP (1 μM), and Rot/Ant (0.5 μM). Quantification of basal respiration, maximal respiration, ATP production, spare respiratory capacity, and non-mitochondrial respiration. All measurements were normalized to cell counts (n=3 cultures). (E) The effect of A-769662 on mitochondrial function was abolished with Compound C. Representative traces showing the OCR of hiPSC-CMs following sequential addition of Oligo (2 μM), FCCP (1 μM), and Rot/Ant (0.5 μM). Quantification of basal respiration, maximal respiration, ATP production, spare respiratory capacity, and non-mitochondrial respiration. All measurements were normalized to cell counts (n=3-4 cultures). Data are presented as mean ± SEM. *P < 0.05, **P < 0.01, ***P < 0.001, ****P < 0.0001 by one-way ANOVA. A100, A-769662 at 100 μM; A200, A-769662 at 200 μM; DMSO, dimethyl sulfoxide; E10, EX229 at 10 μM; E50, EX229 at 50 μM; FCCP, carbonyl cyanide p-(trifluoromethoxy) phenylhydrazone; OCR, oxygen consumption rate; Oligo, oligomycin; Rot/Ant, rotenone/antimycin A; TMRM, tetramethylrhodamine, methyl ester.

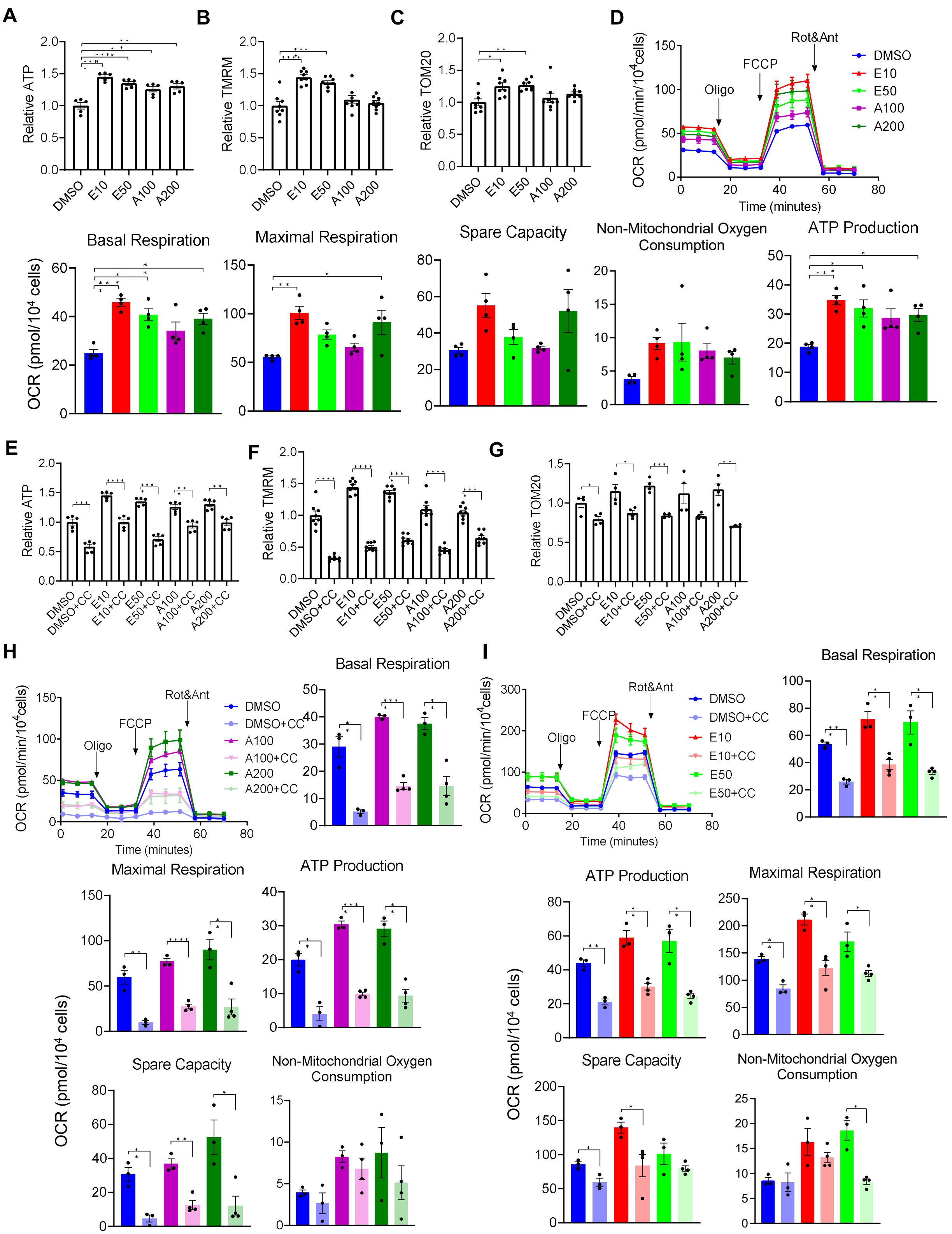
A
Relative ATP
DMSO
E10
E50
A100
A200
B
Relative TMRM
C
Relative TOM20
D
OCR (pmol/min/10^4cells)
Oligo
FCCP
Rot&Ant
Time (minutes)
DMSO
E10
E50
A100
A200
Basal Respiration
Maximal Respiration
Spare Capacity
Non-Mitochondrial Oxygen Consumption
ATP Production
OCR (pmol/10^4 cells)
E
Relative ATP
DMSO
DMSO+CC
E10
E10+CC
E50
E50+CC
A100
A100+CC
A200
A200+CC
F
Relative TMRM
G
Relative TOM20
H
OCR (pmol/min/10^4cells)
Oligo
FCCP
Rot&Ant
Time (minutes)
DMSO
DMSO+CC
A100
A100+CC
A200
A200+CC
Basal Respiration
Maximal Respiration
ATP Production
Spare Capacity
Non-Mitochondrial Oxygen Consumption
OCR (pmol/10^4 cells)
I
OCR (pmol/min/10^4cells)
Oligo
FCCP
Rot&Ant
Time (minutes)
DMSO
DMSO+CC
E10
E10+CC
E50
E50+CC
Basal Respiration
ATP Production
Maximal Respiration
Spare Capacity
Non-Mitochondrial Oxygen Consumption
OCR (pmol/10^4 cells)

Supplementary Figure 6. AMPK activation for 14 days improved metabolic maturation of SCVI273 derived cardiomyocytes. (A) Quantitative analysis of ATP content (n=5 cultures). (B) Quantitative analysis of TMRM by ArrayScan (n=8 cultures). (C) Quantitative analysis of TOM20 by ArrayScan (n=8 cultures). (D) Representative traces of real-time measurement of OCR and quantification of mitochondrial functional parameters including basal respiration, maximal respiration, ATP production, non-mitochondrial respiration, and spare respiratory capacity. All measurements were normalized to cell counts (n=4 cultures). AMPK knockdown using Compound C inhibits the metabolic maturation. Measurements of (E) Relative ATP content (F) Relative TMRM indicating mitochondrial membrane potential (G) Relative TOM20 indicating mitochondrial content (H) The effect of EX229 on mitochondrial function was abolished with Compound C. Representative traces showing the OCR of hiPSC-CMs following sequential addition of Oligo (2 μM), FCCP (1 μM), and Rot/Ant (0.5 μM). Quantification of basal respiration, maximal respiration, ATP production, spare respiratory capacity, and non-mitochondrial respiration. All measurements were normalized to cell counts (n=3-4 cultures). (I) The effect of A-769662 on mitochondrial function was abolished with Compound C. Representative traces showing the OCR of hiPSC-CMs following sequential addition of Oligo (2 μM), FCCP (1 μM), and Rot/Ant (0.5 μM). Quantification of basal respiration, maximal respiration, ATP production, spare respiratory capacity, and non-mitochondrial respiration. All measurements were normalized to cell counts (n=3-4 cultures). Data are presented as mean ± SEM. *P < 0.05, **P < 0.01, ***P < 0.001, ****P < 0.0001 by one-way ANOVA. A100, A-769662 at 100 μM; A200, A-769662 at 200 μM; DMSO, dimethyl sulfoxide; E10, EX229 at 10 μM; E50, EX229 at 50 μM; FCCP, carbonyl cyanide p-(trifluoromethoxy) phenylhydrazone; OCR, oxygen consumption rate; Oligo, oligomycin; Rot/Ant, rotenone/antimycin A; TMRM, tetramethylrhodamine, methyl ester.

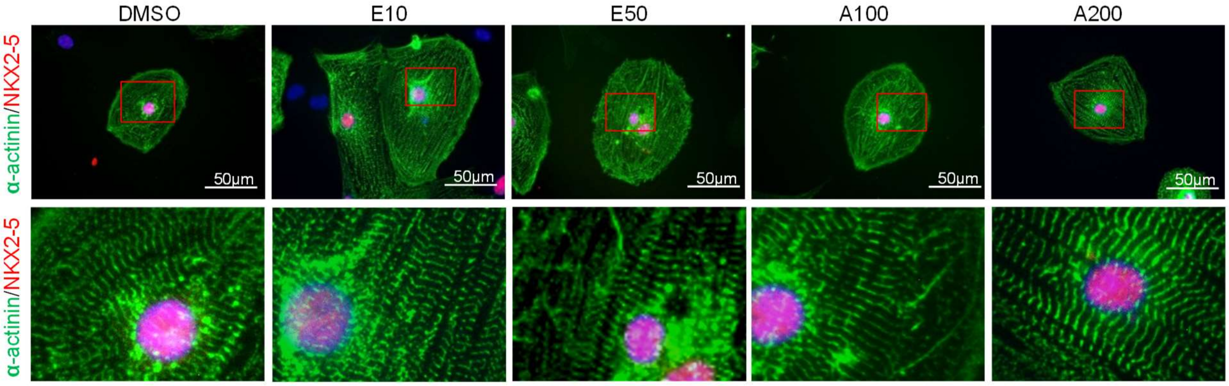

Supplementary Figure 7. Representative images for structural analysis of hiPSC-CMs after 7 days of the maturation treatment. hiPSC-CMs were co-stained with α-actinin and NKX2-5. A100, A-769662 at 100 μM; A200, A-769662 at 200 μM; DMSO, dimethyl sulfoxide; E10, EX229 at 10 μM; E50, EX229 at 50 μM.

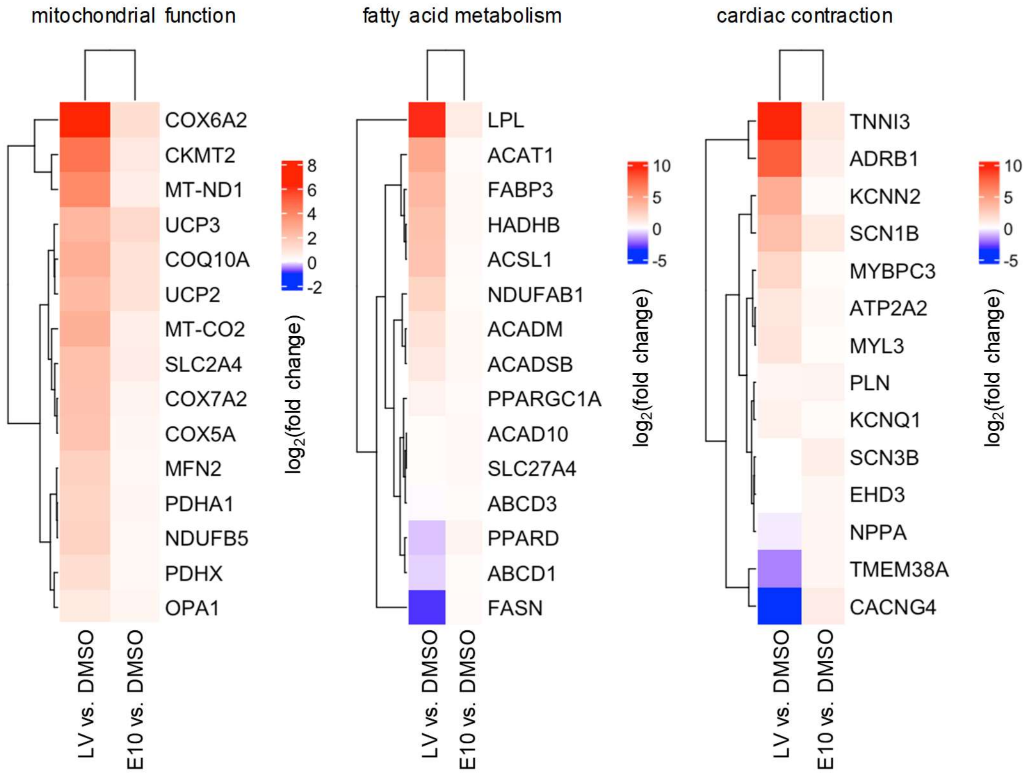

Supplementary Figure 8. Heatmaps showing differentially expressed genes in mitochondrial function, fatty acid metabolism and cardiac contraction in LV vs. DMSO-treated hiPSC-CMs and E10-treated hiPSC-CMs vs. DMSO treated hiPSC-CMs. Log_2(fold changes) were generated from RNA-seq analysis (n=3 cultures or tissue samples). DMSO, dimethyl sulfoxide; E10, EX229 at 10 μM; LV, heart tissue samples from pediatric left ventricle.

Video S1. Cardiac spheres beating activity at Day 7
